# Neonatal but not juvenile gene therapy reduces seizures and prolongs lifespan in *SCN1B*–Dravet syndrome mice

**DOI:** 10.1172/JCI182584

**Published:** 2025-01-23

**Authors:** Chunling Chen, Yukun Yuan, Heather A. O’Malley, Robert Duba-Kiss, Yan Chen, Karl Habig, Yosuke Niibori, Samantha L. Hodges, David R. Hampson, Lori L. Isom

**Affiliations:** 1 Department of Pharmacology, University of Michigan Medical School, Ann Arbor, Michigan, USA.; 2 Department of Pharmacology and Toxicology and; 3 Department of Pharmaceutical Sciences, University of Toronto, Toronto, Ontario, Canada.

**Keywords:** Neuroscience, Therapeutics, Epilepsy, Mouse models, Sodium channels

## Abstract

Dravet syndrome (DS) is a developmental and epileptic encephalopathy (DEE) that begins in the first year of life. While most cases of DS are caused by variants in *SCN1A*, variants in *SCN1B*, encoding voltage-gated sodium channel β1 subunits, are also linked to DS or to the more severe early infantile DEE. Both disorders fall under the OMIM term DEE52. *Scn1b-*null mice model DEE52, with spontaneous generalized seizures and death in 100% of animals in the third postnatal week. *Scn1b-*null cortical parvalbumin-positive interneurons and pyramidal neurons are hypoexcitable. The goal of this study was to develop a proof-of-principle gene replacement strategy for DEE52. We tested an adeno-associated viral vector encoding β1 subunit cDNA (AAV-Navβ1) in *Scn1b-*null mice. We demonstrated that AAV-Navβ1 drives β1 protein expression in excitatory and inhibitory neurons in mouse brains. Bilateral intracerebroventricular administration of AAV-Navβ1 in *Scn1b-*null mice at postnatal day 2 (P2), but not at P10, reduced spontaneous seizure severity and duration, prolonged lifespan, prevented hyperthermia-induced seizures, and restored cortical neuron excitability. AAV-Navβ1 administration to WT mice resulted in β1 overexpression in brain but no obvious adverse effects. This work lays the foundation for future development of a gene therapeutic strategy for patients with *SCN1B*-linked DEE.

## Abstract 2

The goal of this study was to develop a proof-of-principle, adeno-associated viral gene replacement strategy for *SCN1B*-linked developmental and epileptic encephalopathy. This work lays the foundation for future development of a gene therapeutic strategy to treat patients with this devastating disease.

## Graphical abstract

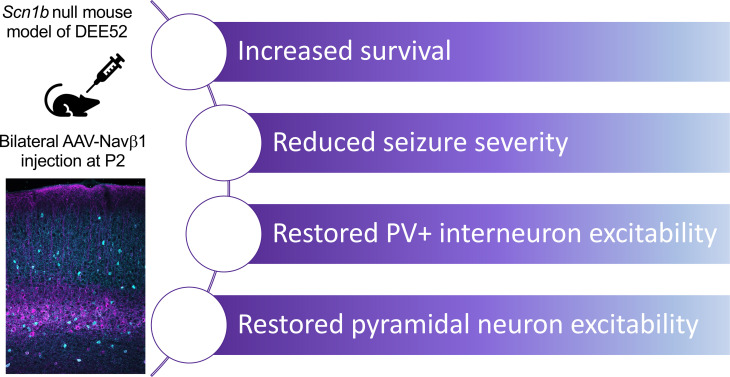

## Introduction

Dravet syndrome (DS) is a devastating developmental and epileptic encephalopathy (DEE) characterized by multiple types of pharmacoresistant seizures beginning in the first year of life, intellectual disability, developmental delay, ataxia, and increased risk of sudden unexpected death in epilepsy (SUDEP) (1, 2). In most cases, DS is caused by de novo pathogenic variants in *SCN1A*, encoding the voltage-gated sodium channel (VGSC) α subunit Nav1.1 (3, 4). Biallelic variants in *SCN1B*, encoding the VGSC non–pore-forming β1 subunit, are also linked to DS or to the more severe early infantile DEE. Both disorders fall under the OMIM term DEE52 (5–10).

VGSCs are essential for mammalian life. They are responsible for generating the rising phase and propagation of action potentials in mammalian excitable cells (11). Purification of mammalian brain VGSCs revealed a central ion-conducting α subunit associated with 2 different non–pore-forming β subunits, β1 or β3 and β2 or β4, encoded by *SCN1B*–*SCN4B*, respectively (11, 12). VGSC β1 subunits are multifunctional molecules that engage in conducting and non-conducting roles in multiple tissues (13). A growing body of evidence has shown the essential role of *SCN1B* in normal physiology as well as in pathophysiology. The presence of an extracellular immunoglobulin (Ig) domain enables β1 subunits to function as Ig superfamily cell adhesion molecules (CAMs) (10, 14, 15). β1 CAM-mediated functions are critical in brain and heart development (10, 14, 16). Ig domain integrity is also critical for β1-mediated sodium current (I_Na_) modulation in vivo (17). As VGSC and voltage-gated potassium channel modulators and plasma membrane chaperones, β1 subunits make important contributions to the regulation of neuronal and cardiac excitability (18–25). As CAM substrates for regulated intramembrane proteolysis (RIP) by β-site amyloid precursor protein–cleaving enzyme-1 (BACE1) and γ-secretase, β1 subunits also contribute to transcriptional regulation (9, 22, 26, 27).

*Scn1b-*null mice model DEE52, with a phenotype that includes spontaneous generalized seizure onset in the second postnatal week, ataxia, failure to gain weight, cardiac arrhythmia, and death in 100% of animals in the third postnatal week (9, 28, 29). *Scn1b*-mediated modulation of excitability is neuronal cell type specific. *Scn1b-*null cortical parvalbumin-positive (PV^+^) fast-spiking (FS) interneurons and cortical pyramidal neurons are hypoexcitable, undergoing depolarization block at high levels of current injection (30), while hippocampal neurons (28, 31), dorsal root ganglion neurons (32), and cerebellar neurons (33, 34) show population-specific excitability changes. We used pharmacological and computational approaches to demonstrate that I_Na_ density heterogeneity between adjacent cortical pyramidal neurons, which normally regulates spike pattern diversity and network synchronization, is impaired in the *Scn1b-*null brain, suggesting that epilepsy develops via promotion of network synchrony (35). The maturation of GABAergic signaling is delayed in the *Scn1b-*null brain. GABA responses remain depolarizing until the time of death in the third postnatal week (29). Noncanonical roles of β1 also contribute to the regulation of excitability. β1 Subunits are posttranslationally modified by Fyn kinase–mediated tyrosine phosphorylation, *S*-palmitoylation, and RIP (36). Palmitoylation of an intracellular cysteine residue near the transmembrane domain is critical for β1 plasma membrane targeting and cleavage via RIP. Following RIP, the released β1 intracellular domain (ICD) translocates to the nucleus to contribute to the regulation of VGSC α subunit mRNA abundance, including *Scn1a*, as well as the mRNA abundance of potassium and calcium channel genes and other signaling molecules (22, 37, 38). The genotype of *Scn1b-*null mice is *Scn1a^+/+^*, yet *Scn1b*-null somatosensory cortical neurons express reduced levels of *Scn1a* mRNA and Nav1.1 protein due to the loss of β1-mediated transcriptional regulation secondary to RIP (9).

The long-term goal of this work is to develop a gene replacement strategy for *SCN1B*, using *Scn1b*-null mice as proof of principle, toward a future gene therapy for DEE52 patients. We tested the effects of an adeno-associated viral (AAV) vector encoding C-terminal epitope–tagged β1 cDNA under control of a version of the *Gad1* promoter (AAV-Navβ1) (39) administered via bilateral intracerebroventricular (i.c.v.) injection to *Scn1b-*null mouse brain at postnatal day 2 (P2). We found that AAV-Navβ1 administration drives β1 protein expression in both excitatory and inhibitory neurons. AAV-Navβ1–treated *Scn1b-*null mice have reduced spontaneous seizure severity and duration in early life, prolongation of lifespan, reduced susceptibility to hyperthermia-induced seizures, elevation of *Scn1a* mRNA expression to WT levels, and restoration of cortical FS PV^+^ interneuron and pyramidal neuron excitability. In contrast, AAV-Navβ1 administration at P10 was ineffective, suggesting that therapeutic intervention at early neonatal time points provides the greatest efficacy. Taken together, this work lays the foundation for future development of a gene therapeutic agent to treat *SCN1B*-linked DEE52.

## Results

### AAV-Navβ1 increases Scn1b and Scn1a mRNA expression and β1 protein in Scn1b-null mouse brain.

**Figure 1:**
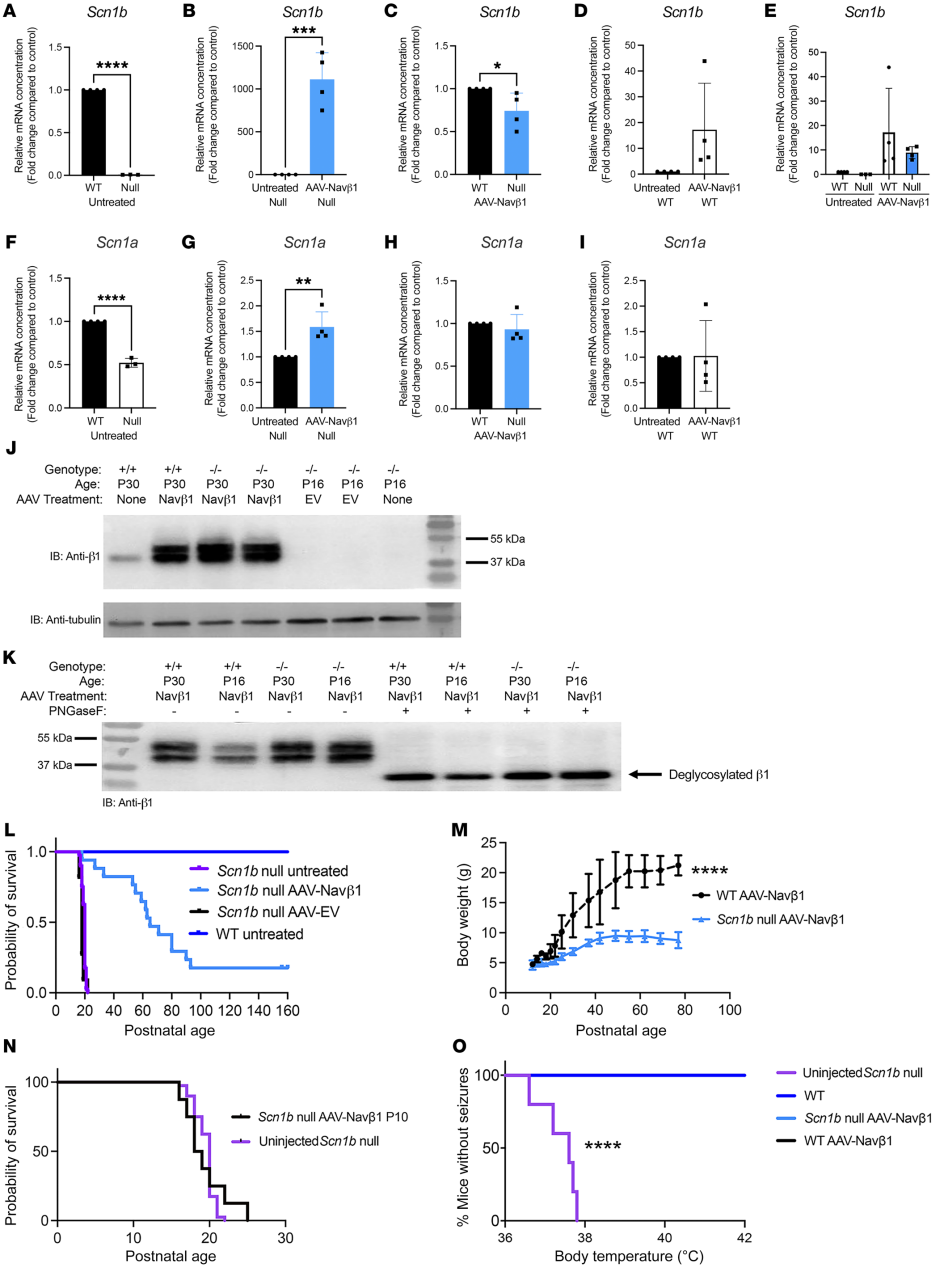
AAV-Navβ1 administration increases *Scn1b* and *Scn1a* mRNA and β1 protein and prolongs lifespan in *Scn1b*-null mice. (**A**) Absence of *Scn1b* mRNA in null somatosensory cortex. ****P < 0.0001. (**B**–**I**) mRNA abundance in P16–18 mouse somatosensory cortex; RT-qPCR data normalized to WT. Data were analyzed using 2-tailed, unpaired *t* test except in **E**, analyzed by 1-way ANOVA. (**B**) *Scn1b* in untreated and AAV-Navβ1–treated null. ***P < 0.005. (**C**) *Scn1b* in AAV-Navβ1–treated P16–18 WT and null. *P < 0.05. (**D**) *Scn1b* in untreated and AAV-Navβ1–treated WT. *P* = 0.1225. (**E**) *Scn1b* in untreated and AAV-Navβ1–treated WT and null. *P* = 0.1003. (**F**) *Scn1a* in untreated WT and null. ****P < 0.0001. (**G**) *Scn1a* in untreated and AAV-Navβ1–treated null. **P < 0.0074. (**H**) *Scn1a* in AAV-Navβ1–treated WT and null. *P* = 0.4626. (**I**) *Scn1a* in untreated versus AAV-Navβ1–treated WT. *P* = 0.9431. (**J**) Western blot analysis of WT (+/+) or null (–/–) mouse brain. Immunoblot (IB): Anti-β1. Bottom: IB of the same blot with anti-tubulin. (**K**) Western blot analysis of AAV-Navβ1–treated WT (+/+) or null (–/–) brains with or without PNGase F treatment. IB: Anti-β1. Arrow, deglycosylated β1 immunoreactive bands. (**L**) Single i.c.v. dose of AAV-Navβ1 at P2 improved survival of null (light blue) versus untreated (purple) or AAV-EV–treated null mice (black) through P160 (*P* < 0.0001, log-rank Mantel-Cox test). Dark blue, untreated WT mice. Kaplan-Meier (Wilcoxon) analysis. (**M**) Single i.c.v. dose of AAV-Navβ1 at P2 did not increase body weights of null (solid blue line) versus WT mice (dashed black line) through P80 (****P < 0.0001, 2-way ANOVA). (**N**) Single i.c.v. dose of AAV-Navβ1 at P10 had no effect on null (black) versus untreated null mouse survival (purple; data taken from **L**) (log-rank Mantel-Cox test). (**O**) Single i.c.v. dose of AAV-Navβ1 at P2 prevented hyperthermia-induced seizures in P16 null mice. Kaplan-Meier analysis presented for first observed Racine scale 5–6 seizure; uninjected null (purple, *n* = 5), uninjected WT (dark blue, *n* = 5), AAV-Navβ1–injected null (light blue, *n* = 6), or AAV-Navβ1–injected WT mice (black, *n* = 5). ****P < 0.0001.

We examined the effect of bilateral i.c.v. AAV-Navβ1 administration at P2 (1.1 × 10^11^ to 1.7 × 10^11^ vector genomes per mouse) on *Scn1b* mRNA abundance in mouse brain somatosensory cortex measured at P16–18 using quantitative reverse transcriptase PCR (RT-qPCR). We confirmed the absence of *Scn1b* mRNA in untreated *Scn1b*-null somatosensory cortex at P16–18 (Figure 1A). Administration of AAV-Navβ1 at P2 resulted in significantly increased *Scn1b* mRNA abundance (Figure 1B). Comparison of *Scn1b* mRNA abundance in AAV-Navβ1–treated WT and null mice showed that, as expected, levels in WT were significantly greater than in nulls (Figure 1C). We compared *Scn1b* mRNA abundance in untreated and AAV-Navβ1–treated nulls (Figure 1D). The high variability in the treated levels between mice resulted in no significant differences between groups, but the trend suggested an increase in treated animals. Finally, we compared *Scn1b* mRNA abundance in untreated and AAV-Navβ1–treated WT nulls in order to plot the values on the same scale (Figure 1E). As expected, the increase in AAV-treated brains was much larger than that in untreated, although as in Figure 1D, the high variability in the treated levels between mice resulted in no significant differences between groups.

In previous work, we showed that *Scn1b*-null mice have reduced expression of *Scn1a* mRNA and Nav1.1 protein in somatosensory cortex and hippocampus, despite their *Scn1a^+/+^* genotype, suggesting an additive mechanism for the severity of the *Scn1b*-null model via disrupted regulation of another gene critical in DS (9, 28). Figure 1F confirms that result. Here, we asked whether AAV-Navβ1 treatment at P2 restored *Scn1a* mRNA abundance in *Scn1b*-null somatosensory cortex to WT levels. Comparison of *Scn1a* mRNA abundance in untreated versus AAV-Navβ1–treated *Scn1b*-null samples showed a significant increase in treated animals (Figure 1G). *Scn1a* mRNA abundance was similar in AAV-Navβ1–treated WT versus AAV-Navβ1–treated *Scn1b*-null somatosensory cortex (Figure 1H). Finally, *Scn1a* mRNA abundance in AAV-Navβ1–treated *Scn1b*-null somatosensory cortex was not significantly different from that in untreated WT (Figure 1I). Taken together, these results support the hypothesis that the cleaved ICD generated from WT β1 polypeptides regulates *Scn1a* mRNA abundance in mouse brain and, in its absence, *Scn1a* mRNA is reduced (22). Replacement of β1 protein restores *Scn1a* expression in mouse brain.

Bilateral i.c.v. AAV-Navβ1 administration at P2 resulted in overexpression of β1 polypeptides in *Scn1b*-null brain. We performed Western blot analysis of untreated WT, AAV-Navβ1–treated null, and untreated null whole brain membrane preparations at P30 or P16 as indicated, using an anti-β1 antibody recognizing the β1-ICD (9) (Figure 1J, top). Expression of β1 was absent in null brain treated with AAV-EV (empty vector control), as in untreated null brain. Anti-tubulin antibody was used as a control for sample loading (Figure 1J, bottom). Anti-β1 antibody detected multiple immunoreactive bands at and above 37 kDa in the AAV-Navβ1–treated mouse brain samples (Figure 1, J and K). Deglycosylation of brain samples using PNGase F collapsed these bands to a single species running at the predicted molecular weight of native, unglycosylated β1 (~28 kDa; Figure 1K, arrow), demonstrating differential glycosylation of β1 polypeptides in vivo in agreement with previous data (9, 17, 40, 41).

### AAV-Navβ1 treatment at P2 prolongs Scn1b-null mouse lifespan.

Kaplan-Meier analysis of mouse survival confirmed that 100% of untreated *Scn1b*-null animals underwent premature death in the third postnatal week (28, 29) (Figure 1L, purple). WT mice lived normal lifespans (Figure 1L, dark blue). WT mice injected with AAV-Navβ1 at P2 showed no toxicity and lived normal lifespans (21 WT pups injected during the course of the study lived until culled for other experiments at variable time points, some for >100 days; not shown), consistent with previous work (39). Bilateral i.c.v. administration of AAV-Navβ1 at P2 (1.1 × 10^11^ to 1.7 × 10^11^ vector genomes per mouse) dramatically extended null mouse lifespan (Figure 1L, light blue), with some animals living longer than P100, when they were culled for experimentation (*P* < 0.0001, log-rank Mantel-Cox test), so this graph underestimates their survival. In contrast, bilateral i.c.v. administration of AAV-EV at P2 had no effect on null mouse lifespan (Figure 1L, black).

*Scn1b*-null mice fail to thrive, with small stature and low body weight compared with WT littermates (28, 29). Untreated null mouse body weights plateau after the second postnatal week and rarely increase past 5.5 g until the time of death at about P21 (29). P19–21 AAV-Navβ1–treated null mice weighed approximately 5 g (Figure 1M). Despite the remarkable prolongation of null mouse lifespan resulting from AAV-Navβ1 administration in a subset of mice, body weights did not exceed 10 g measured up to the age of P80 (Figure 1M, solid blue line). In contrast, AAV-Navβ1–treated WT mice reached a normal weight of approximately 20 g by approximately P40, similar to that reported previously for untreated WT mice (29) (*P* < 0.0001, 2-way ANOVA; Figure 1M, dashed black line).

### AAV-Navβ1 administration at P10 has no effect on Scn1b-null mouse lifespan.

We injected 3 litters of *Scn1b* pups with AAV-β1 at P10 to achieve a more valid clinical temporal scenario. Five WT and 8 null pups each received an AAV-Navβ1 dose of 3.4 × 10^11^ vector genomes, twice the dose administered to P2-injected mice. While the WT pups showed no toxicity in terms of seizures or mortality, all 8 null pups died between P16 and P25, the usual time frame during which untreated null mice die. Kaplan-Meier analysis comparing the survival of P10-injected null mice with untreated null mice (untreated null mouse data reproduced from Figure 1L) is shown in Figure 1N. Thus, i.c.v. administration of AAV-Navβ1 to null mice at P10 was ineffective. Because of this result, all remaining experiments were performed on animals injected at P2 (1.1 × 10^11^ to 1.7 × 10^11^ vector genomes per mouse).

### AAV-Navβ1 reduces early-life seizure severity in Scn1b-null mice.

One hundred percent of *Scn1b*-null mice exhibit spontaneous, generalized seizures during the second postnatal week that result in death in the third postnatal week (9, 28, 29). We conducted continuous infrared video monitoring to compare seizure onset, frequency, and severity in AAV-Navβ1–treated (injected at P2) and untreated null mice. Recordings began at P14 owing to difficulties in visualizing pups beneath the dam before this time, combined with the feasibility of the tagging and genotyping process. The small stature and fragility of null mice precluded surgical implantation of EEG electrodes, regardless of AAV treatment. Each mouse video was analyzed offline separately by 3 investigators blinded to treatment using the modified Racine scale (42), with only grade 5–6 seizures counted and timed owing to the challenge of assessing lower-grade seizures in mouse pups. Results were compared and discrepancies resolved between investigators by referring to the video together.

Supplemental Video 1 shows clips of 3 untreated *Scn1b*-null pups monitored from P14 through P16. We observed grade 5–6 seizures during the first day of recording for each pup, consistent with ref. 9 and ref. 28. We counted an average of 16.4 spontaneous, generalized seizure-like events less than 10 seconds in duration as well as 8.7 spontaneous, generalized seizure-like events of greater than 10 seconds, and up to 2 minutes, in duration per day per pup from P14 through P16 (Table 1). Supplemental Video 2 shows clips of 3 untreated null mice that experienced SUDEP following a terminal seizure during the third week of life (P21 to P24). The terminal SUDEP events in null mice were similar to those shown for (C57BL/6J × 129S6/SvEvTac)F1 *Scn1a^+/–^* DS mice, including tonic hind-limb extension at 180° to the torso (43).

Supplemental Video 3 shows three P2-injected AAV-Navβ1–treated *Scn1b*-null pups monitored from P14 through P16. In contrast to untreated animals, no spontaneous generalized seizure-like events lasting more than 10 seconds were observed for any of the 3 mice during this period (Table 1). Brief loss-of-posture events that appeared to be seizures less than 10 seconds in duration were observed 3–8 times per day per animal beginning on the first day of recording, with an average of 8 events per day per animal, suggesting that seizure severity is reduced, but time to first seizure is not altered, by AAV-Navβ1 treatment (Table 1). Supplemental Video 3 shows 10 separate episodes of brief seizure-like events with loss of posture from three P14–16 AAV-Navβ1–treated mice. Without electrographic evidence, we cannot be confident that these events were indeed seizures. Nevertheless, we observed far fewer seizure-like events that were less severe in AAV-Navβ1–treated null animals compared with the untreated cohort.

We next analyzed infrared videos of *Scn1b*-null pups from 10 litters that received bilateral AAV-Navβ1 injections at P2. This cohort included 40 null animals that lived past P30. Three of the 40 animals were observed to die of SUDEP between P59 and P70 (Supplemental Video 4, which includes 3 video clips). Other animals had wasting deaths, but most were culled for experimentation during this time range, so their time of natural death was unknown. The terminal generalized seizures in the mice that experienced SUDEP were visually similar in severity and phenotype, with hind-limb extension, to those recorded in untreated null mice (Supplemental Video 2). However, in contrast to untreated mice, these long-lived, AAV-Navβ1–treated animals were observed to have only mild, brief seizure-like events during the recording period, similar to those shown in Supplemental Video 3, up until the terminal seizure (not shown).

Finally, we analyzed 3 consecutive days of infrared video recordings from the 5 AAV-Navβ1–treated *Scn1b*-null mice that lived to P100 before being culled for use in other experiments. Note that only 5 mice were in this group because of the use of all others for experiments. Supplemental Video 5 consists of 2 clips: The first shows three P100 AAV-Navβ1–treated null mice. The second clip shows two P101 AAV-Navβ1–treated null mice plus an AAV-Navβ1–treated P101 WT mouse from the same litter for size comparison. No generalized seizure-like events, including short seizure-like events, were observed in any of the AAV-Navβ1–treated null or WT mice during the recording period. Other than their smaller size in comparison with WT littermates, AAV-Navβ1–treated null mice were visually indistinguishable from WT. Thus, while the effects of AAV-Navβ1 administration at P2 are variable between individual animals, this treatment can dramatically reduce seizure severity and increase lifespan in a subset of null mice.

We tested automated seizure detection software to increase the statistical power of our study with a larger sample size; however, these programs did not reliably detect the shorter seizures. The analyses presented required manual scoring of 648 hours of video recordings per individual (1,944 hours total). A limitation of our study was that we could not afford the time and personnel to analyze additional animals.

### AAV-Navβ1 treatment prevents hyperthermia-induced seizures in Scn1b-null mice.

Pediatric DEE52 patients have frequent febrile seizures (9, 44). *Scn1a^+/–^* neonatal mice (45), which model DS, and *Scn1b^+/–^* neonatal mice (17), which model genetic epilepsy with febrile seizures plus, have increased susceptibility to hyperthermia-induced seizures. Using an experimental design similar to that in ref. 9, we tested untreated and P2–AAV-Navβ1–treated *Scn1b*-null and WT mice for hyperthermia-induced seizure susceptibility at P16 (Figure 1O). We show that 5 of 5 untreated null pups had Racine grade 5–6 seizures ending in SUDEP between body temperatures of 37.2°C and 37.7°C. In contrast, 0 of 6 AAV-Navβ1–treated null pups as well as 0 of 5 AAV-Navβ1–treated WT pups showed Racine grade 5–6 seizures during the experimental testing period up to a body temperature of 42°C.

### Cellular and temporal specificity of AAV-Navβ1 expression in Scn1b-null mouse brain.

We assessed the distribution of AAV-Navβ1 expression in the brains of P2-injected pups at P30 by immunostaining using anti-myc antibody and found the CNS distribution to be similar to that in ref. 39. Navβ1-myc–positive signal was detected in the forebrains of both WT and *Scn1b-*null mice given AAV-Navβ1, whereas expression in most posterior regions, such as the midbrain and brain stem, was not detected (Supplemental Figure 1, B and C). No Navβ1-myc transgene expression was observed in WT mice given empty vector AAV-EV (Supplemental Figure 1A).

Confocal microscopy confirmed the expression of Navβ1-myc in the forebrain; strong and dense myc-positive signal was seen in the somatosensory cortex (Supplemental Figure 2A) and the pyramidal layer of the hippocampus (Supplemental Figure 2C), with comparatively weaker and sparser signal in the striatum (Supplemental Figure 2B). Sparse amounts of immunopositive cells were also detected in the granule layer of the cerebellum (Supplemental Figure 2D).

Higher magnification images of Navβ1-myc–immunopositive cells are shown for the somatosensory cortex (Supplemental Figure 3, A–C, gray), the caudoputamen of the striatum (Supplemental Figure 3, D–F, gray), the pyramidal cell layer of the hippocampal CA3 region (Supplemental Figure 3, G–I, gray), and the granule layer of the cerebellum (Supplemental Figure 3, J–L, gray) of WT mice treated with AAV-EV or AAV-Navβ1 or null mice treated with AAV-Navβ1, as indicated. Navβ1-myc–positive signal was localized to the cellular somata and processes in all regions examined. In all panels, DAPI (shown in cyan) was used as a nuclear marker. Additionally, immunopositive cells throughout the cerebellar granule layer were found to overlap with large DAPI^+^ nuclei, suggesting that they may be GABAergic Golgi neurons (46) (Supplemental Figure 3, J–L).

Quantitative double-label immunohistochemistry was performed in the somatosensory cortices of P2-injected AAV-Navβ1–treated WT or *Scn1b-*null mice at P30 to assess the cell type selectivity of expression (Supplemental Figure 4). Anti-NeuN was used as a general neuronal marker, anti-GABA was used to broadly label all GABAergic neurons, anti-PV was used to label FS interneurons, and anti-somatostatin (anti-SST) or anti–vasoactive intestinal polypeptide (anti-VIP) was used to label SST- or VIP-expressing interneurons, respectively. Anti-myc signals were mainly restricted to cortical NeuN^+^ neurons in both WT and *Scn1b-*null mice (87.4% and 83.8%, respectively). A total of 34.4% of NeuN^+^ neurons in WT mice and 26.4% in null mice were transduced (Supplemental Figure 4, A, B, and I). GABAergic neuron specificity and coverage were similar in both genotypes (10.9% in WT and 10.8% in null; coverage 22.4% WT and 14.8% null) (Supplemental Figure 4, C, D, and J). Of the GABAergic neuron subtypes, anti-myc expression was detected in PV^+^, SST^+^, and VIP^+^ interneurons (Supplemental Figure 4, E–J). PV interneuron specificity was 7.9% for both genotypes, with 45.9% coverage in WT and 36.0% in null mice (Supplemental Figure 4M). In contrast, SST specificity was lower overall (2.8% in WT and 2.9% in null), although a higher proportion of SST^+^ interneurons were transduced (63.8% in WT and 50.6% in null) (Supplemental Figure 4N). VIP specificity (0.35% in WT and 0.72% in null) and coverage (7.75% in WT and 17.72% in null) were both low (Supplemental Figure 4O). Finally, we confirmed that AAV-Navβ1 protein expression was not detectable in microglia (Supplemental Figure 5, Iba1) or astrocytes (Supplemental Figure 5, GFAP) in either WT (top panels) or null (bottom panels) mice.

**Figure 2:**
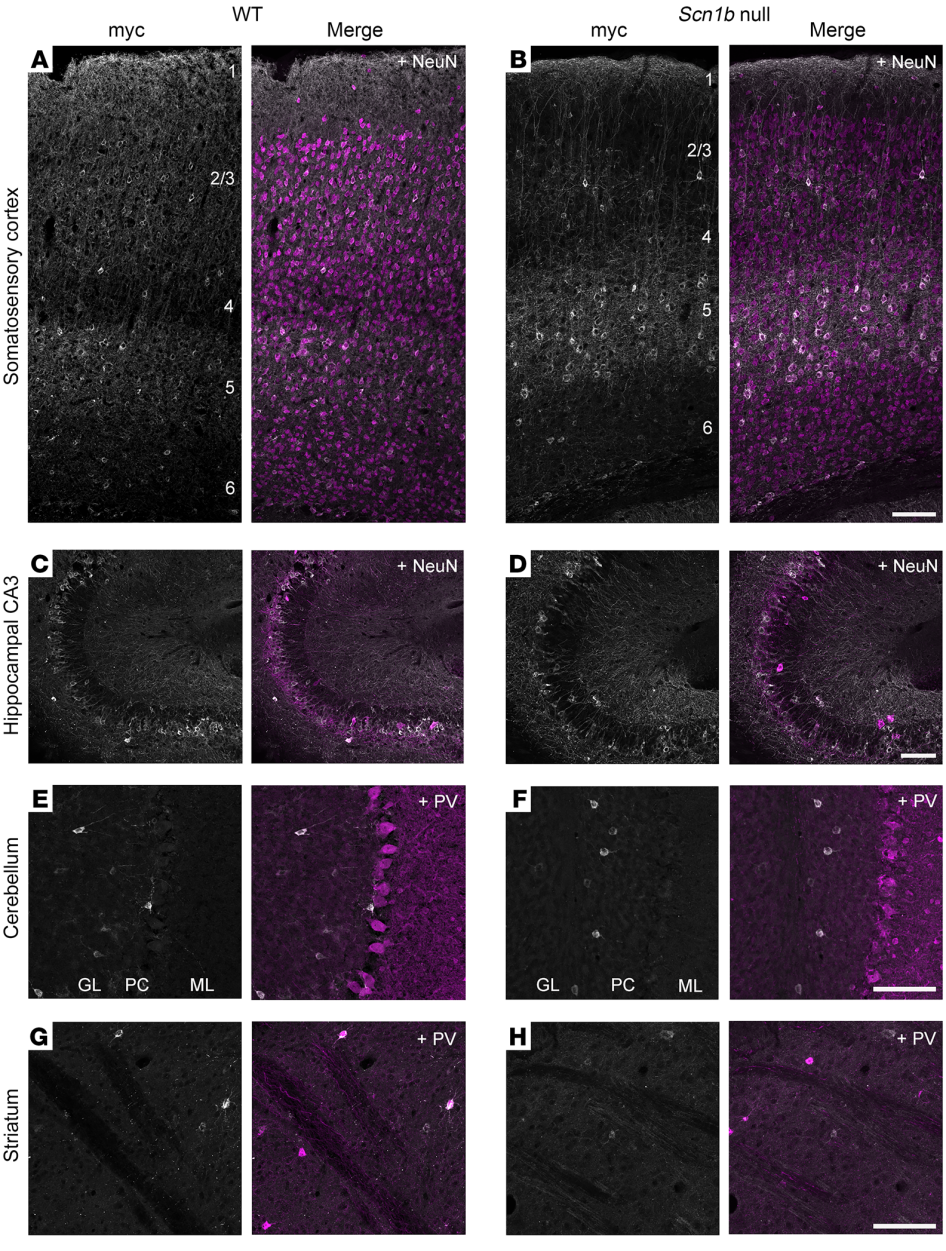
Navβ1-myc is variably expressed in brain regions at P135 and P160. (**A** and **B**) Navβ1-myc expression in layers 1–6 of somatosensory cortex of WT (**A**) or null (**B**) mice. Gray, myc; merge, myc plus NeuN (magenta). (**C** and **D**) Navβ1-myc is expressed primarily in the pyramidal cell layer in hippocampus, with CA3 displayed here, of WT (**C**) or null (**D**) mice. Gray, myc; merge, myc plus NeuN (magenta). (**E** and **F**) Navβ1-myc expression in scattered cells within the cerebellar granule layer of WT (**E**) or null (**F**) mice. Gray, myc; merge, myc plus PV (magenta); GL, granule cell layer; PC, Purkinje cell layer; ML, molecular layer. (**G** and **H**) Navβ1-myc expression was minimal in striatum (caudoputamen) of WT (**G**) or null (**H**) mice. Gray, myc; merge, myc plus PV (magenta). Scale bars: 100 μm.

We next examined the expression of Navβ1-myc protein in P2-injected mice at P135 for WT and P160 for *Scn1b*-null mice that were each culled for experimentation (Figure 2). At P135, Navβ1-myc was expressed in all layers of the somatosensory cortex in WT animals (Figure 2A). In contrast, expression in P160 null cortex was largely restricted to layer 5, with sparse neuronal labeling in other layers, which was different from the primarily homogeneous expression in this brain region of null animals observed at P30 (Supplemental Figure 2A). Interestingly, we observed a distinct columnar labeling pattern in null layers 2–3 at P160 (Figure 2B). Navβ1-myc showed abundant expression in hippocampal CA3 in both genotypes (Figure 2, C and D). In the cerebellum, as at P30, Navβ1-myc expression was restricted to a small number of neurons within the granule cell layer in both genotypes (Figure 2, E and F). Very few labeled neurons were detected in striatum in either genotype (Figure 2, G and H). Examination of low-magnification sagittal sections and coordinating high-resolution confocal images of AAV-Navβ1–treated P91–160 WT and null mice that were culled for experimentation also revealed Navβ1-myc protein in the cerebellar fastigial nucleus, the vestibular nuclei of the brainstem, and the pontine region, brain areas in which anti-myc immune signal was less prominent at P30 (Supplemental Figure 6).

The lack of effect of P10 AAV-Navβ1 administration on *Scn1b*-null mouse survival could reflect developmental changes in the null brain at this time point that cannot be reversed by gene replacement or alternatively could reflect reduced viral coverage in the brain despite the increased dosage. Previous work demonstrated that viral administration to mouse brain at P0–2 provides superior diffusion of the vector within the brain compared with P5 and that efficacy dramatically decreases with increasing age (47). We examined expression of Navβ1-myc protein in FS PV^+^ interneurons and GABAergic neurons in P10-injected mice using quantitative double-label immunohistochemistry. Because mortality in null pups begins at P16, we chose this age to visualize distribution of Navβ1-myc in P10-injected WT and null mice. We also imaged Navβ1-myc expression in WT and *Scn1b^+/–^* mice at P38, 4 weeks after viral injection (Supplemental Figure 7, A–D). Specificity and coverage in both neuronal subtypes were similar between genotypes (PV: specificity 14.0% WT, 17.4% *Scn1b^+/–^*, and coverage 79.4% WT, 77.7% *Scn1b^+/–^*; GABA: specificity 23.6% WT, 29.3% *Scn1b^+/–^*, and coverage 68.0% WT, 71.0% *Scn1b^+/–^*) (Supplemental Figure 7, E–H). Consistent with previous work (47), the efficacy of viral administration at P10 was decreased in comparison with P2. Brain expression of Navβ1-myc protein in P10-injected animals was substantially restricted in comparison with P2-injected animals. For P10-injected animals imaged at both P16 and P38, a limited number of neurons in cortex and hippocampus expressed Navβ1-myc, with no expression detected in frontal cortex or cerebellum (Supplemental Figure 7, I–L). We found a high degree of variability in the extent of Navβ1-myc expression in P10-injected null and WT mice imaged at P16, with some animals showing nearly no Navβ1-myc signal, which precluded our ability to quantify expression at this time point.

### AAV-Navβ1 restores Scn1b-null PV^+^ interneuron and pyramidal neuron excitability.

**Figure 3:**
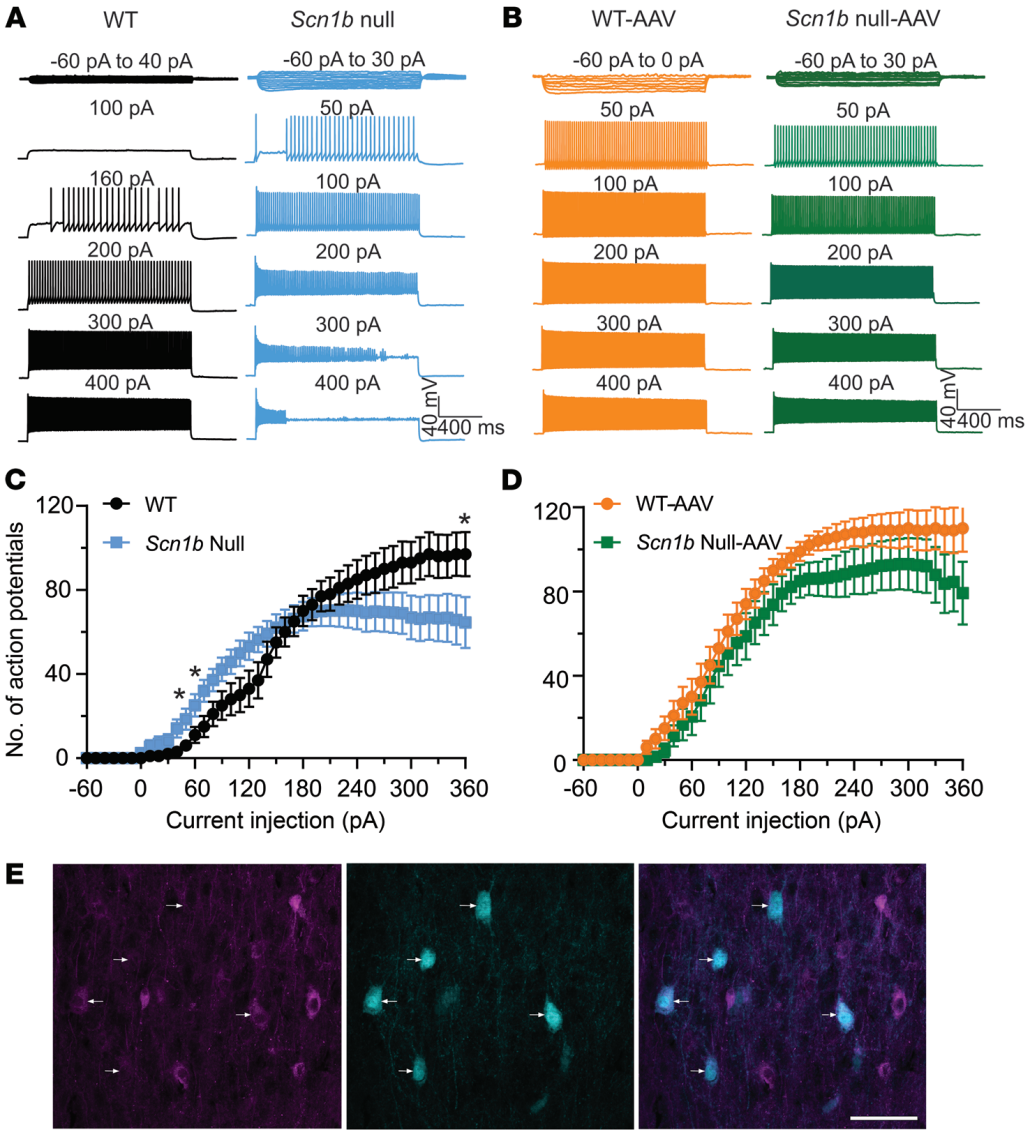
AAV-Navβ1 restores *Scn1b*-null PV^+^ interneuron excitability. (**A**) Representative traces showing evoked repetitive firing of untreated PV^+^ interneurons in cortical layer 2/3 brain slices from P16–18 WT (black) or null (blue) mice. Repetitive AP firing was evoked by injections of 1,500-millisecond currents from –60 pA to 330 pA at 10-pA steps from RMP. A representative null interneuron began to fire APs at lower intensities of current injection compared with the WT. Stronger depolarizing current injections blocked repetitive firing in the null interneuron. (**B**) Representative traces showing evoked repetitive firing of cortical layer 2/3 PV^+^ interneurons in slices from P16–18 WT (orange) or null (green) mice following a single dose of AAV-Navβ1 at P2. WT and null interneurons showed similar AP firing patterns in response to current injections. (**C**) I-O curves for AP firing of untreated WT (black) versus null (blue) PV^+^ interneurons in response to current injections. I-O curves were generated by plotting of the number of APs evoked by 1,500-millisecond current injections against current intensities over a range of –60 pA to 330 pA. Asterisks denote significant differences between genotypes (*P* < 0.05). (**D**) I-O curves for AP firing of WT (orange) versus null (green) PV^+^ interneurons following AAV-Navβ1 treatment. Values are mean ± SEM of 13 cells from 7 untreated WT mice, 17 cells from 6 AAV-treated WT mice, 18 cells from 7 untreated null mice, or 12 cells from 6 AAV-treated null mice. No significant differences between genotypes. (**E**) tdTomato-labeled PV^+^ neurons (cyan) in P17 somatosensory cortex of AAV-treated *Scn1b*^–/–^/PV-Cre/tdTomato mice show variable expression of AAV-Navβ1 (magenta) among PV^+^ neurons. Arrows, myc antibody labeling (left) in PV^+^ neurons (middle) with merged image (right). Scale bar: 50 μm.

*Scn1b*-null cortical PV^+^ interneurons show hypoexcitability at high current injections (30). To determine whether AAV-Navβ1 treatment of null mice impacts PV^+^ interneuron excitability, we used *Scn1b^+/–^*/PV-Cre/tdTomato mice on the C57BL/6J background, as in previous work (30), to facilitate visualization of PV^+^ neurons by epifluorescence for recording. We administered AAV-Navβ1 to entire litters of mice at P2 and then compared the action potential (AP) firing properties of WT and null PV^+^ interneurons in the somatosensory cortical regions of acute brain slices at P16–18. Representative traces recorded from untreated WT (black) or null (blue) cortical PV^+^ interneurons in brain slices are shown in Figure 3A. Representative AP firing patterns of P16–18 WT (orange) and null PV^+^ (green) interneurons following a single, bilateral i.c.v. dose of AAV-Navβ1 at P2 are shown in Figure 3B. Untreated null interneurons began to fire at lower current injections than untreated WT. At higher current injections, untreated null neurons decreased their firing rate, while untreated WT neurons continued to fire.

Input-output (I-O) curves for AP firing in response to current injections for all recorded PV^+^ cells from untreated null and WT animals are summarized in Figure 3C. Null PV^+^ interneurons required less depolarizing current injection to initiate AP firing compared with WT. They fired significantly more APs in the lower range of depolarizing current injection than WT, indicating hyperexcitability. However, as current injection intensities increased, null interneurons fired significantly fewer APs and became sensitive to depolarization-induced block compared with WT interneurons. Others observed a similar biphasic firing pattern in *Scn1a^+/–^* DS mice in previous work, suggesting common disease mechanisms (48). Interestingly, for *Scn1b* mice, we found 2 subpopulations of PV^+^ interneurons that responded to depolarizing current injections with different thresholds to generate APs. In our pooled data we observed a deflection in the I-O curve between 80 pA and 140 pA (Figure 3C). Upon further analysis, we were able to separate the data into 2 groups (Supplemental Figure 8A). One subpopulation of PV^+^ interneurons (9 of 15 cells) began to generate APs in response to 20 to 60 pA current injections. The remaining 6 of 15 WT PV^+^ interneurons did not generate APs until injections of 100 to 150 pA currents. While the I-O curve for pooled null PV^+^ cells (Figure 3C) was smoother than that for WT, we also found 2 subpopulations of null PV^+^ interneurons that responded to current injections with different thresholds (Supplemental Figure 8B). Comparison of these subpopulations of null PV^+^ interneurons with their corresponding WT subpopulations showed that both needed smaller depolarizing current injection intensities to evoke AP firing (Supplemental Figure 8, C and D).

We compared I-O curves of AP firing for AAV-Navβ1–treated WT (orange) versus AAV-Navβ1–treated null (green) PV^+^ interneurons and found no significant differences between genotypes (Figure 3D). Interestingly, AAV-Navβ1 administration changed the firing patterns of both WT and null neurons. While AAV-Navβ1 treatment reversed the depolarization block observed in null neurons, it did not alter the shape of the I-O curve at lower current injections. In contrast, we observed a leftward, hyperpolarizing, shift in the I-O curve for AAV-Navβ1–treated WT PV^+^ neurons, with increased AP firing at lower current injections. The mechanism of this shift is not known, although we hypothesize that it may be due to the extraordinarily high levels of β1 protein in the cortex resulting from AAV-Navβ1 administration on top of normal *Scn1b* expression. High levels of β1 overexpression may increase the rate of I_Na_ activation or shift the voltage dependence of I_Na_ activation in the hyperpolarizing direction, resulting in a reduced threshold for AP initiation (25, 49). Nevertheless, despite this hyperpolarizing shift in WT PV^+^ neuronal firing, AAV-Navβ1–treated WT animals did not display spontaneous seizures or premature death.

Importantly, AAV-Navβ1 administration eliminated the depolarization block observed in untreated null PV^+^ neurons. We found it interesting that, while quantification of anti-myc immunofluorescence in AAV-Navβ1–treated brain slices showed overlap with only a subset of anti-PV^+^ neurons (Supplemental Figure 4), our electrophysiological experiments showed that virtually all tdTomato^+^ null neurons recorded under epifluorescence showed restored AP firing. Our previous work showed a high degree of overlap between tdTomato^+^ epifluorescence and anti-PV^+^ staining in *Scn1b^–/–^*/PV-Cre/tdTomato mice (30), indicating that this method of PV^+^ neuron identification in brain slices has a high degree of fidelity. The overlap of tdTomato^+^ epifluorescence (red) and anti-myc^+^ staining (green) in *Scn1b^–/–^*/PV-Cre/tdTomato mouse brain slices is shown in Figure 3E. Consistent with Supplemental Figure 4, only a subset of tdTomato^+^ neurons is anti-myc^+^. We interpret these results, in agreement with previous work in heterologous cells (25, 49, 50), to mean that very little *Scn1b* mRNA is required to produce sufficient β1 protein to fully modulate I_Na_ and that the antibodies used for immunofluorescence detection do not have sufficient sensitivity and/or affinity to allow visualization of every cell that was expressing Navβ1-myc.

The AP properties of P16–18 WT versus null untreated and AAV-Navβ1–treated PV^+^ interneurons are compared in Supplemental Figure 9, with results summarized in Supplemental Table 1. Untreated null PV^+^ interneurons had significantly reduced resting membrane potential (RMP), reduced minimum current required for AP initiation, reduced AP maximal rise rate, and reduced peak AP amplitude compared with WT, in agreement with previous work showing that the loss of β1 decreases I_Na_ density in PV^+^ interneurons. These differences were resolved following AAV-Navβ1 treatment. There were no significant differences in cell capacitance, AP threshold potential, AP half-width, or AP maximum decay rate between groups.

**Figure 4:**
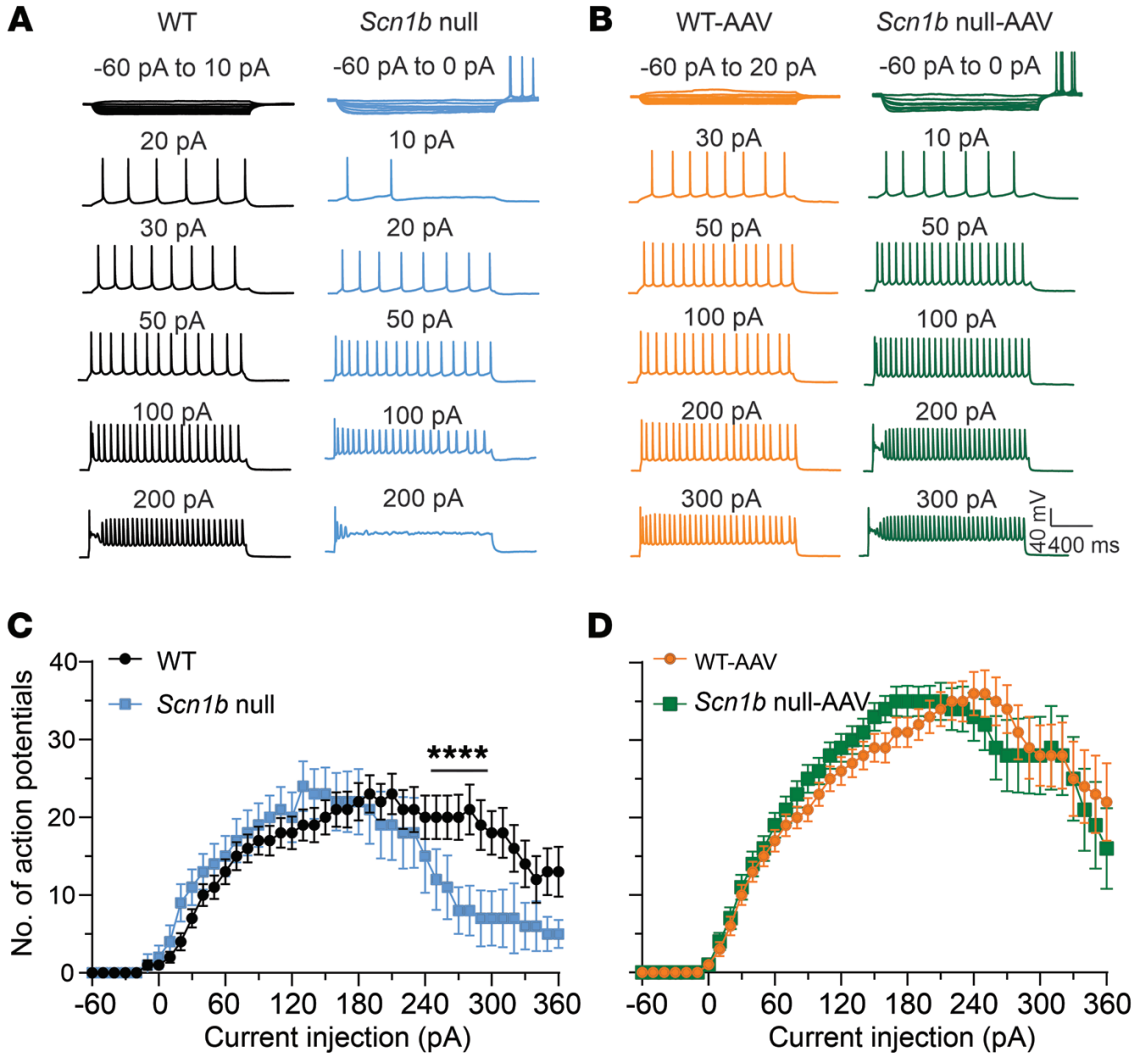
AAV-Navβ1 restores *Scn1b*-null pyramidal neuron excitability. (**A**) Representative traces showing evoked repetitive firing of cortical pyramidal neurons of brain slices from untreated WT (black) or null (blue) mice. Repetitive AP firing was evoked by injections of 1,500-millisecond currents from –60 pA to 330 pA (selected responses are shown) at 10-pA steps from RMP. (**B**) Representative traces showing evoked repetitive firing of cortical pyramidal neurons of brain slices from AAV-Navβ1–treated WT (orange) or null (green) mice. Repetitive AP firing was evoked by injections of 1,500-millisecond currents from –60 pA to 330 pA (selected responses are shown) at 10-pA steps from RMP. (**C**) I-O curves for AP firing of untreated WT versus null pyramidal neurons in response to current injections. I-O curves were generated by plotting of the number of APs evoked by 1,500-millisecond current injections against current intensities over a range of –60 pA to 330 pA. Values are mean ± SEM of 23 cells from 8 untreated WT mice, 19 cells from 7 AAV-treated WT mice, 15 cells from 4 untreated null mice, or 19 cells from 8 AAV-treated null mice. Asterisks denote significant differences between genotypes (*P* < 0.0001). (**D**) I-O curves for AP firing of AAV-Navβ1–treated WT versus null pyramidal neurons in response to current injections. I-O curves were generated by plotting of the number of APs evoked by 1,500-millisecond current injections against current intensities over a range of –60 pA to 330 pA. No significant differences between genotypes.

*Scn1b* affects the excitability of pyramidal neurons in addition to PV^+^ interneurons (30, 31). Thus, pyramidal neurons are also predicted to be impacted by AAV-Navβ1 administration. To test this hypothesis, we compared the AP firing properties of WT and null pyramidal neurons, identified using infrared differential interference contrast (IR-DIC) optics, in somatosensory cortical layers 2–6 of acute brain slices at P16–18. Depolarization block in the AP firing pattern and cumulative I-O curves for untreated null pyramidal neurons (blue) compared with WT (black) is shown in Figure 4. AAV-Navβ1 administration at P2 resulted in similar AP firing patterns between genotypes recorded at P16–18 (Figure 4, C and D). Comparison of the AP properties of P16–18 WT versus null untreated and AAV-Navβ1–treated pyramidal neurons is shown in Supplemental Figure 10, with results summarized in Supplemental Table 2. Untreated null pyramidal neurons had significantly increased input resistance, reduced peak AP amplitude, and reduced maximum AP maximal rise rate compared with WT, in agreement with previous work showing that the loss of β1 decreases I_Na_ density in pyramidal neurons. These differences were resolved following AAV-Navβ1 treatment. We found no significant differences in RMP, cell capacitance, threshold potential, AP half-width, or maximum AP decay rate between groups (Supplemental Table 2). Taken together, these results show that AAV-Navβ1 administration restores AP firing properties of both excitatory and inhibitory neurons in mouse brain.

## Discussion

Monoallelic variants in *SCN1B* are linked to genetic epilepsy with febrile seizures plus (GEFS+) (10, 17). Biallelic variants in *SCN1B* are linked to DEE52, which can be diagnosed as DS (5, 6, 44) or the more severe early infantile DEE (7–9). Dysregulation of *SCN1B* gene expression is also proposed to contribute to cortical excitation/inhibition imbalance in autism spectrum disorder (51, 52), which is a significant comorbidity of DEE52 (44). While seizures are the most obvious outcomes of DEE, other aspects of the disease, including profound developmental delay and intellectual disability, impact quality of life as much as, if not more than, seizures (44). Evidence suggests that, because DEE genes like *SCN1B* can independently impact brain development, the “developmental” and “epileptic” aspects of DEE may be separable; thus small-molecule drugs that target seizures often have little or no effect on developmental comorbidities caused by gene disruption (53). Clearly, there is an unmet clinical need for the discovery of novel therapeutic tools that target the genetic basis of DEE52.

Our previous work demonstrated the efficacy of an antisense oligonucleotide (ASO) targeting a nonsense-mediated decay (NMD) exon in *SCN1A* to overcome Nav1.1 haploinsufficiency in a mouse model of DS (54). Because NMD exons have not been identified in *SCN1B*, and because DEE52 patients express 2 mutant *SCN1B* alleles that result in the translation of mutant polypeptides, this ASO strategy (55) may not be appropriate for the *SCN1B* patient population. Here, we tested a gene replacement approach using an AAV9 vector to overexpress VGSC β1 cDNA in mouse brain. AAV-Navβ1, which drives β1 expression in central excitatory and inhibitory neurons, was previously developed as a potential treatment for *SCN1A*-linked DS, based on our work showing that β1 subunits function as plasma membrane chaperones and channel modulators of VGSC α subunits (25, 39). AAV-Navβ1 administration provided only moderate therapeutic benefit in *Scn1a*-linked DS mice, whereas in the present study the AAV-Navβ1–generated transgene served as a direct replacement for this missing protein. Because *Scn1b* deletion impacts the firing properties of both excitatory and inhibitory neurons in mice (30), we reasoned that AAV-Navβ1 may be more effective in the *Scn1b*-null DEE mouse model. Here, we show that bilateral i.c.v. administration of AAV-Navβ1 to null mice at P2 results in restoration of *Scn1b* mRNA and β1 protein expression in the brain, reduced seizure severity in early life, prolongation of lifespan, prevention of hyperthermia-induced seizures, restoration of *Scn1a* mRNA expression in the somatosensory cortex, and restoration of cortical PV^+^ interneuron and pyramidal neuron excitability.

A key treatment outcome, the significant extension of lifespan following P2-administered AAV gene therapy, was not observed in mice injected at P10. We attribute this effect to the relatively low level and distribution of the Navβ1 transgene in comparison with P2-injected mice. Previous work has shown that 7–14 days is required after administration of single-stranded AAV vectors to achieve therapeutic levels of expression in rodent brains (56); thus Navβ1 levels in null mice were likely insufficient during the period of seizure onset (P10–13) to prevent SUDEP. Other AAV gene therapy studies (47, 57), as well as our unpublished observations with gene therapy for fragile X syndrome (Y. Niibori, A.W.M. Hooper, D.R. Hampson), have found dramatically reduced AAV transgene CNS expression with injection later than P3–4 in mice and rats. In the context of AAV-mediated gene therapy, it remains to be established whether this phenomenon occurs in human infants and, if so, during what developmental window. Nevertheless, recessive DEE gene variants like *SCN1B*, which can be diagnosed in utero via genetic testing, provide the opportunity for early, neonatal intervention.

In addition to the brain, VGSCs are expressed in enteric neurons, smooth muscle cells, and interstitial cells of Cajal within the gastrointestinal (GI) tract (58–60). Thus, it is not surprising that DS patients, including those with *SCN1B* gene variants, present with comorbid GI symptoms, including feeding difficulties, constipation, and failure to thrive (8, 61). *Scn1b*-null mice also fail to thrive, with plateau of body weight at approximately P10 (28, 29). Diet supplementation with gel food on the floor of the cage, treatment with bumetanide (29), or administration of AAV-Navβ1 does not result in significant weight gain (Figure 1). Anecdotally, *Scn1b*-null mice consume gel food more rapidly and in larger amounts than age-matched WT littermates, yet their bodies do not grow accordingly. *Scn1b*-null mice have reduced glucose-stimulated insulin and glucagon secretion from pancreatic islets, in vitro and in vivo, suggesting an important role for VGSC β1 in mouse pancreatic glucose homeostasis (62). We reported previously in abstract form that, despite their stagnation in body weight, *Scn1b*-null mouse small intestines continue to increase in length with age, perhaps as a compensatory mechanism (63). In addition, null mice have reduced total muscle mass and reduced intestinal lipid absorption (63). In future work, it will be interesting to administer AAV-Navβ1 peripherally, with a different promoter, to investigate the role of *Scn1b* in pancreatic function, GI tract formation and function, enteric neuron signaling processes, and gut-brain axis transmission.

While the *Scn1b*-null mouse model phenocopies many aspects of DEE52, patients are not null for *SCN1B*. Except for one variant located in a *SCN1B* splice acceptor site (c.449-2A>G) that may result in aberrant splicing of *SCN1B* mRNA to delete transmembrane β1 polypeptides (8), *SCN1B* DEE variants generate β1 polypeptides that are expressed in cellular membranes and differentially modulate I_Na_ in heterologous cells (reviewed in ref. 10). Two *SCN1B* variants, p.C121W and p.R89C, have been expressed in vivo using transgenic mouse knockin strategies (9, 17, 64). The phenotype of homozygous *Scn1b*-p.C121W mice is similar to that of *Scn1b*-null mice, with reduced size, spontaneous generalized seizures, and 100% mortality (64). Heterozygous *Scn1b*-p.C121W mice, which model GEFS+, are more susceptible to hyperthermia-induced seizures than *Scn1b* heterozygous or WT mice. Biochemical, immunofluorescence, and electrophysiological data suggested that *SCN1B-*p.C121W may confer deleterious gain of function and compete with WT β1 subunits in heterozygous animals (17, 64). The recessive variant, *SCN1B*-p.R89C, has been identified in 3 families with children diagnosed with DS/DEE52 (9, 65). Monoallelic parents are asymptomatic. Homozygous *Scn1b*-p.R89C knockin mice have normal body weights and approximately 20% premature mortality, which is markedly different from *Scn1b-*null mice (9). Similarly to *Scn1b*-null mice, 100% of homozygous *Scn1b*-p.R89C knockin mice have spontaneous generalized seizures and are more susceptible to hyperthermia-induced seizures compared with WT. Heterologous expression data suggested that the *SCN1B-*p.R89C variant results in partial loss of function but, in contrast to *SCN1B*-p.C121W, does not exert deleterious gain-of-function effects in the presence of WT β1. Would viral overexpression of WT β1 be beneficial to DEE52 patients with *SCN1B* variants? An essential next step in therapeutic development will be to test the efficacy of AAV-Navβ1 in *Scn1b*-p.C121W and *Scn1b*-p.R89C knockin mice or in c.449-2A>G patient-derived induced pluripotent stem cell neurons. It may be that some, but not all, of the effects of *SCN1B* DEE variants can be overcome by overexpression of WT β1 in brain and that this gene replacement strategy may not be appropriate, or safe, for all *SCN1B* patients. Nevertheless, the development of AAV-Navβ1 is a major step forward toward the goal of gene replacement therapy for *SCN1B*-linked DEE.

## Methods

### Sex as a biological variable.

Approximately equal numbers of male and female mouse pups were used in all experiments.

### Animals.

Investigators were blinded to genotype for all experiments. Animals were housed in the Unit for Laboratory Animal Medicine at the University of Michigan Medical School. *Scn1b^–/–^* (null), *Scn1b^+/–^* (Het), and *Scn1b^+/+^* (WT) littermate mice were generated and genotyped as previously described (28) and were congenic on the C57BL/6J background for over 30 *N* generations. To label PV^+^ FS neurons in null brains for patch clamp electrophysiology, *Scn1b^+/–^* mice were crossed with PV-Cre/tdTomato mice on the C57BL/6J background to generate *Scn1b^+/+^/*PV-Cre/tdTomato and *Scn1b^–/–^/*PV-Cre/tdTomato mice as previously described (9, 30).

### AAV vector generation and virus production.

The pAAV9-pGad1-Navβ1-myc (AAV-Navβ1) vector consisted of a truncated mouse *Gad1* promoter, mouse Navβ1 cDNA with C-terminal in-frame myc and FLAG epitope tags, a woodchuck hepatitis virus posttranslational regulatory element (WPRE), and a rabbit β-globin polyadenylation region (39). AAV empty vector (AAV-EV), consisting of a cytomegalovirus promoter, WPRE, and rabbit β-globin poly(A) region flanked by inverted terminal repeats, was used as a control (39). AAV-Navβ1 and AAV-EV (both serotype 9) vectors were produced at the University of Pennsylvania Vector Core Facility (Philadelphia, Pennsylvania, USA) as fee-for-service. Several batches of AAV-Navβ1 were used with titers ranging from 5.3 × 10^13^ to 8.5 × 10^13^ genome copies/mL.

### AAV administration.

All pups from *Scn1b^+/–^* breeding pair litters were injected with AAV-EV or AAV-Navβ1, as indicated, via bilateral i.c.v. injection of 1 μL into each hemisphere at P2 or 2 μL into each hemisphere at P10, as indicated, using a Hamilton syringe, as in ref. 54. Thus, the dose given was between 1.1 × 10^11^ and 1.7 × 10^11^ vector genomes per mouse for the P2-injected animals or 2.2 × 10^11^ and 3.4 × 10^11^ vector genomes per mouse for the P10-injected animals.

### Survival analysis and video monitoring.

Videos were recorded using Omniplex D software and hardware (Plexon) and securely stored under password protection. Videos were recorded continuously starting at P14 with infrared illumination during the dark cycle. Cameras were oriented to provide full field of view of the observation chamber to ensure that generalized seizures would not be occluded and that terminal events could be captured accurately. Mice that died during the study were removed from the arena during daily husbandry checks. Other mice were culled at specific time points for experimental analyses, as indicated. Each video was viewed separately and scored manually for seizures using the modified Racine scale (42) by 3 investigators blinded to genotype and treatment. Survival data were compiled for each treatment and genotype in GraphPad Prism 10.0 software using Kaplan-Meier (Wilcoxon) analysis.

### Hyperthermia-induced seizures.

Hyperthermia seizure susceptibility in untreated and P2–AAV-β1–treated null mice was tested at P16 (9, 17). Seizures were classified according to a modified Racine scale (6, 17, 42). After a 1 mL intraperitoneal injection of 0.9% NaCl to prevent dehydration, a rectal thermometer was positioned to monitor body temperature (BT). A heat lamp connected to a temperature monitoring system controlled BT. Mice were acclimated in the chamber at 37.5°C for 30 minutes. During the observation period, the set temperature (ST) was increased by 0.5°C and then held for 2 minutes. At the approximately 25-minute time point, ST was held at 42°C for an additional 15 minutes. When a seizure was observed, BT, seizure severity (Racine scale), and time elapsed from the beginning of the observation period were recorded. Animals that did not undergo SUDEP were euthanized at the end of the experiment. Investigators were blinded to genotype.

### Western blot analyses.

Mouse brain membrane proteins were prepared (25) at ages indicated in the figure legends. Complete protease inhibitor cocktail (Roche Diagnostics) was added to all solutions at twice the recommended concentration to minimize protein degradation. Deglycosylation of membrane protein samples was performed using PNGase F (New England BioLabs P0704S) (17). Fifty- to eighty-microgram aliquots of membrane protein were separated by 10% SDS-PAGE and processed for Western blotting with anti-β1_intra_ antibody (1:1,000; Cell Signaling Technologies 13950). Anti–α-tubulin antibody (1:1,000; Cedarlane CLX135AP) was used to control for equal sample loading. Immunoreactive bands were detected using SuperSignal West Dura Extended Duration Substrate (Thermo Fisher Scientific 34076) and imaged using an iBrightFL1000 system (Invitrogen).

### RT-qPCR.

RNA was isolated from P15–17 mouse somatosensory cortex using the QIAGEN RNeasy Plus kit according to the manufacturer’s instructions. Tissue was homogenized with a Tissue-Tearor (BioSpec Products Inc.) followed by lysis through a sterile, 18-gauge needle and vortexing. RNA samples were analyzed on a NanoDrop One Spectrophotometer (Thermo Fisher Scientific) for concentration and purity and stored at –80°C. cDNA was generated from 1 μg of RNA using Reverse Transcriptase SuperScript III (RT SS III; Thermo Fisher Scientific), random primers (Invitrogen), and dNTPs (Invitrogen). RNA, random primers, and dNTPs were incubated at 65°C for 5 minutes. Salt buffers, 0.1 M DTT, RNase Out (Invitrogen), and RT SS III were added, and reactions were incubated at 25°C for 5 minutes, 50°C for 60 minutes, and 70°C for 15 minutes. Quantitative PCR was performed using SYBR Green (Applied Biosystems) and gene-specific primers (*Scn1a*, *Scn1b*, *Gapdh*; Integrated DNA Technologies) on a QuantStudio 7 Flex Real-Time PCR System (Applied Biosystems). Gene-specific measurements of each cDNA sample were run in triplicate, along with *Gapdh* for normalization, and compared with WT expression levels. The relative abundance levels of mRNA for each gene were quantified using the comparative threshold (2^–ΔΔCt^) method. Sample sizes for each gene were *n* = 3–4 per group. Data are presented as fold change in gene expression ± SEM. Statistical significance (*P* < 0.05) of comparisons between genotypes was determined using 2-tailed Student’s *t* test.

### Immunohistochemistry.

Mice were anesthetized with isoflurane and transcardially perfused with phosphate-buffered saline (PBS) followed by 4% paraformaldehyde. Brains were dissected, postfixed overnight in 4% paraformaldehyde, sequentially submerged overnight in 10% and 30% sucrose, then flash-frozen in OCT compound and stored at –80°C. Twenty- to thirty-micrometer sagittal sections were generated on a Leica CM1850 cryostat and stored at –20°C until use.

For Figure 2 and Supplemental Figure 5, immunofluorescence labeling was performed as previously described (30, 54). Briefly, slides were rehydrated in 0.05 M phosphate buffer (PB) and blocked for at least 2 hours in blocking buffer (10% normal goat serum and 0.3% Triton X-100 in 0.1 M PB). Slides were incubated in primary antibodies in blocking buffer overnight at room temperature. The next day, slides were washed for 10 minutes 3 times with 0.1 M PB, incubated with secondary antibodies in blocking buffer for 2 hours, washed for 10 minutes 3 times in 0.1 M PB, then mounted with ProLong Gold (Invitrogen) plus DAPI and stored at 4°C until image acquisition. For anti–VGSC β1, sections were incubated in 1% SDS for 5 minutes before blocking. For Supplemental Figure 6, whole-brain images were obtained using a ×10 objective on a Nikon Ti2E epifluorescence microscope with a Nikon Qi2 camera. Tiled images were assembled using Nikon NIS-Elements software.

Primary antibodies used were as follows: rabbit (1:500; Abcam ab9106) or mouse (1:500; Invitrogen MA1-980, clone 9E10) anti–c-myc, guinea pig anti-parvalbumin (1:500; Synaptic Systems 195 004), rabbit anti-VIP (1:500; Cell Signaling Technology D8J1V, 63269), guinea pig anti-NeuN (1:500; Invitrogen ABN90P), rabbit anti–VGSC β1 (1:250; Cell Signaling Technology D9T5B, 14684), mouse anti-GFAP (1:500; Invitrogen 14-9892-82, clone GA5), and rabbit anti-Iba1 (1:500; Fujifilm Wako 019-19741). Secondary antibodies used were as follows: Alexa Fluor goat anti-rabbit, goat anti-mouse, or goat anti–guinea pig antibodies (Invitrogen) conjugated to 488 or 594 nm fluorophores as appropriate.

Fluorescent images were acquired on a Nikon A1R confocal system with a Nikon FN1 microscope at the University of Michigan Department of Pharmacology using a ×20/0.75 NA objective and NIS-Elements AR software. Images were acquired at matched locations relative to midline in somatosensory cortex. Three images from each of 4 mice were analyzed using NIH ImageJ. Graphs were generated and statistical analyses performed using GraphPad Prism 9.4. Figures were assembled in Adobe Photoshop 2023.

For Supplemental Figures 1–4, sections were washed twice with PBS, permeabilized in 1% Triton X-100 in PBS for 30 minutes, and washed. Sections were blocked in 5% bovine serum albumin (Bioshop) plus 5% normal donkey serum (Sigma-Aldrich) in PBS. Sections were incubated overnight in primary antibodies in blocking buffer at 4°C. Sections were washed with PBS and incubated in secondary antibodies diluted in 5% normal donkey serum in PBS for 2 hours. Sections were washed in PBS for 10 minutes 5 times, incubated with 5 μg/mL DAPI, mounted with ProLong Gold Antifade solution, and stored in the dark at 4°C until image acquisition.

For GABA immunolabeling, sections were incubated in rabbit anti-GABA primary antibodies and donkey anti-rabbit Alexa Fluor 594 secondary antibodies, then washed as above. Anti-myc antibodies were conjugated to Alexa Fluor 488 fluorophores using the Alexa Fluor 488 antibody labeling kit (Thermo Fisher Scientific), and sections were incubated overnight at 4°C in conjugated antibodies diluted 1:1,000 in blocking buffer. Sections were then washed for 10 minutes 5 times with PBS, incubated with 5 μg/mL DAPI, and mounted as above.

Primary antibodies used were as follows: rabbit anti-myc (1:4,000; Abcam, ab9106), mouse anti-NeuN (1:2,000; MilliporeSigma, MAB377), rabbit anti-GABA (1:1,000; Sigma-Aldrich, A20502), mouse anti-PV (1:1,000; Sigma-Aldrich, P3088), and rat anti-SST (1:100; MilliporeSigma, MAB354). Secondary antibodies used were as follows: donkey anti–rabbit Alexa Fluor 594 (1:2,000; Invitrogen, A21207), goat anti–mouse Alexa Fluor 488 (1:2,000; Invitrogen, A11029), and goat anti–rat Alexa Fluor 647 (1:3,000; Invitrogen, A21247).

Low-magnification images were acquired using the ×4 objective lens of a Cytation 5 slide scanner (Bio-Rad; in the Center for Pharmaceutical Oncology, Leslie Dan Faculty of Pharmacy, University of Toronto). High-magnification images of Navβ1-myc–expressing cells and images for cell selectivity quantification were taken using a Zeiss LSM 700 confocal microscope (×20 and ×63 magnification). Cell counts were performed by gating of background signal in ImageJ Fiji and manual counting of immunopositive cells using the Cell Counter plug-in. Cell type specificity was calculated as the number of cells double-positive for myc and the cell type marker divided by the total number of myc-positive cells, multiplied by 100%. Cell type coverage was calculated as the number of cells double-positive for myc and the cell type marker divided by the total number of cell type marker–positive cells, multiplied by 100%.

### Brain slice preparation.

Acute brain slices were prepared (31). Mice were anesthetized with isoflurane and decapitated. Brains were removed and placed in 95%:5% O_2_/CO_2_ continuously aerated ice-cold slice solution containing in mM: 110 sucrose, 62.5 NaCl, 2.5 KCl, 6 MgCl_2_, 1.25 KH_2_PO_4_, 26 NaHCO_3_, 0.5 CaCl_2_, and 20 d-glucose (pH 7.35–7.40 when aerated at room temperature). Brains were blocked, and 300-μm-thick coronal sections were obtained from somatosensory cortical areas. Slices were incubated in an aerated holding chamber containing slice solution for 30 minutes at room temperature and then incubated in 1:1 slice/artificial cerebrospinal solution (ACSF) for 30 minutes. ACSF contained in mM: 125 NaCl, 2.5 KCl, 1 MgCl_2_, 1.25 KH_2_PO_4_, 26 NaHCO_3_, 2 CaCl_2_, and 20 d-glucose (pH 7.35–7.40 with aeration). Slices were transferred to an aerated holding chamber containing 100% ACSF for at least 30 minutes before use.

### Electrophysiological recording and analysis.

Individual brain slices were placed in a recording chamber and superfused with 2–3 mL/min aerated ACSF. Pyramidal neurons were identified based on size, shape, and location using a Nikon E600FN upright microscope equipped with IR-DIC optics with a Nomarski 40× water immersion objective. Only vertically oriented pyramidal cells were selected for recording. FS interneurons were identified via red epifluorescence using PV-Cre/tdTomato mice. Recording electrodes had a resistance of 3–6 MΩ with solutions containing in mM: 140 K-gluconate, 4 NaCl, 0.5 CaCl_2_, 10 HEPES, 5 EGTA, 5 phosphocreatine, 2 Mg-ATP, and 0.4 GTP (pH adjusted to 7.2–7.3 with KOH). The junction potential was calculated to be 14.3 mV using the P-clamp junction potential calculator, and all values were corrected offline, with all values presented as corrected values. Following break-in at –94.3 mV in voltage clamp mode, RMP was defined as the membrane potential in current clamp less than 10 seconds after initial break-in. Repetitive firing was elicited in whole-cell current clamp configuration from RMP in 1-second-long current injections in 10-pA steps. There was a 1-second-long 0 current injection period between each sweep. Data were acquired at 20 kHz and filtered at 10 kHz. Cells with an access resistance measured in voltage clamp greater than 20 MΩ or with RMP more depolarized than –64.3 mV were discarded. Access resistance and pipette capacitance were compensated using bridge balance. Cell capacitance was measured using P-clamp whole-cell capacitance compensation in voltage clamp with 10 mV depolarizing steps from –94.3 mV. Automated AP quantification was performed using custom MATLAB (MathWorks) software. APs were defined as the voltage crossing 0 mV subsequent to a dv/dt greater than 10 mV/ms, defined here as the AP threshold. Input resistance was calculated using Ohm’s law with –10 pA current injection from the RMP after 250 ms.

### Statistics.

Comparisons of 2 groups were performed using a 2-tailed, unpaired *t* test. Welch’s correction was applied when the variance between 2 groups was unequal. All data are presented as the mean ± SEM. Data with *P* < 0.05 were deemed significant.

### Study approval.

All animal procedures were performed in accordance with NIH policy and approved by the University of Michigan Institutional Animal Care and Use Committee (PRO00010562).

### Data availability.

Data are available in the Supporting Data Values file or upon request.

## Author contributions

CC and YY contributed equally to this work. They are listed in alphabetical order. CC developed the mouse model; administered AAVs; supervised animal breeding, genotyping, and husbandry; recorded and analyzed mouse spontaneous and hyperthermia-induced seizures, survival, and weight; performed and analyzed Western blot analysis, and wrote the manuscript. YY performed and analyzed all electrophysiological experiments and wrote the manuscript. HAO and RDK performed and analyzed the immunofluorescence experiments and wrote the manuscript. YC performed and analyzed the hyperthermia-induced seizure experiments. YC and KH assisted CC. YN developed the AAV vectors and wrote the manuscript. SLH performed and analyzed the RT-qPCR experiments and wrote the manuscript. DRH and LLI designed and supervised the study, wrote and approved the entire manuscript, and provided funding.

## Supplementary Material

Supplemental data

Unedited blot and gel images

Supplemental video 1

Supplemental video 2

Supplemental video 3

Supplemental video 4

Supplemental video 5

Supporting data values

## Figures and Tables

**Table 1:**
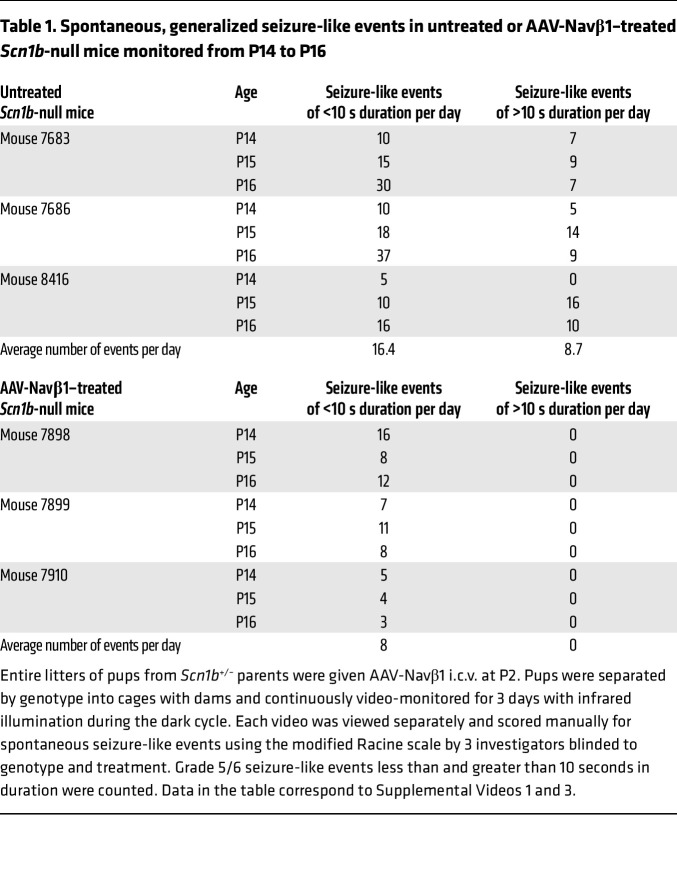
Spontaneous, generalized seizure-like events in untreated or AAV-Navβ1–treated *Scn1b*-null mice monitored from P14 to P16

## Version 1 01/23/2025

In-Press Preview

## Version 2 03/03/2025

Electronic publication
