# Supplementary material for: Neonatal but not juvenile gene therapy reduces seizures and prolongs lifespan in SCN1B–Dravet syndrome mice
Source: J Clin Invest. 2025 Jan 23;135(5):e182584. doi: 10.1172/JCI182584 (PMC11870736; doi:10.1172/JCI182584)
Supplement: Supplemental data [file jci-135-182584-s231.pdf]

SUPPLEMENTAL MATERIAL

Neonatal but not Juvenile Gene Therapy Reduces Seizures and Prolongs Lifespan in *SCN1B*-Dravet Syndrome Mice

Chunling Chen[†1], Yukun Yuan[†1], Heather A. O'Malley[1], Robert Duba-Kiss[2], Yan Chen[1], Karl Habig[1], Yosuke Niibori[3], Samantha L. Hodges[1], David R. Hampson[2,3], and Lori L. Isom*[1]

[†]**These authors contributed equally to this work.**

Author affiliations:

[1]Department of Pharmacology, University of Michigan Medical School, Ann Arbor, MI 48109, USA

[2]Department of Pharmacology and Toxicology, University of Toronto, Toronto, Ontario, Canada

[3]Department of Pharmaceutical Sciences, University of Toronto, Toronto, Ontario, Canada

Correspondence to: Lori L. Isom, Ph.D.

Department of Pharmacology, University of Michigan, 2301 E MSRB III, Ann Arbor, MI 48109-5632, USA

E-mail: lisom@umich.edu

Running title: Gene Therapy for Epilepsy

Keywords: sodium channel, β subunit, epilepsy, Dravet syndrome, AAV9

Conflict of Interest Statement: The authors have declared that no conflict of interest exists.

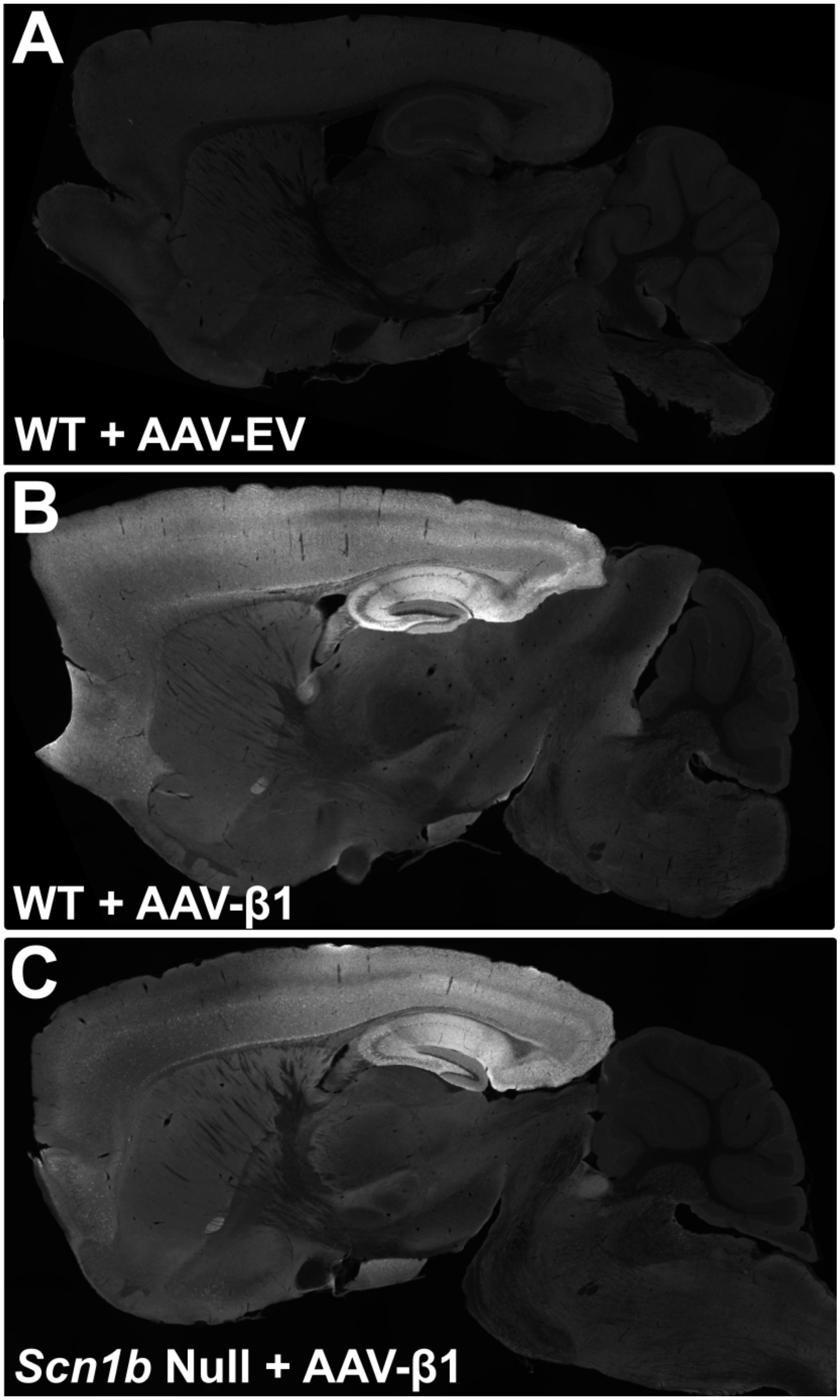

Supplemental Figure 1. Distribution of expression of Navβ1-myc in mouse brain following neonatal AAV administration. Immunofluorescence staining using anti-myc antibody was used to detect transgenic Navβ1-myc in P30 WT mice treated with AAV-EV **(A)** or with AAV-Navβ1 **(B)**, and in P30 *Scn1b* null mice treated with AAV-Navβ1 **(C)** at P2. Image acquisition was performed using 4x magnification on a Cytation5 slide scanner.

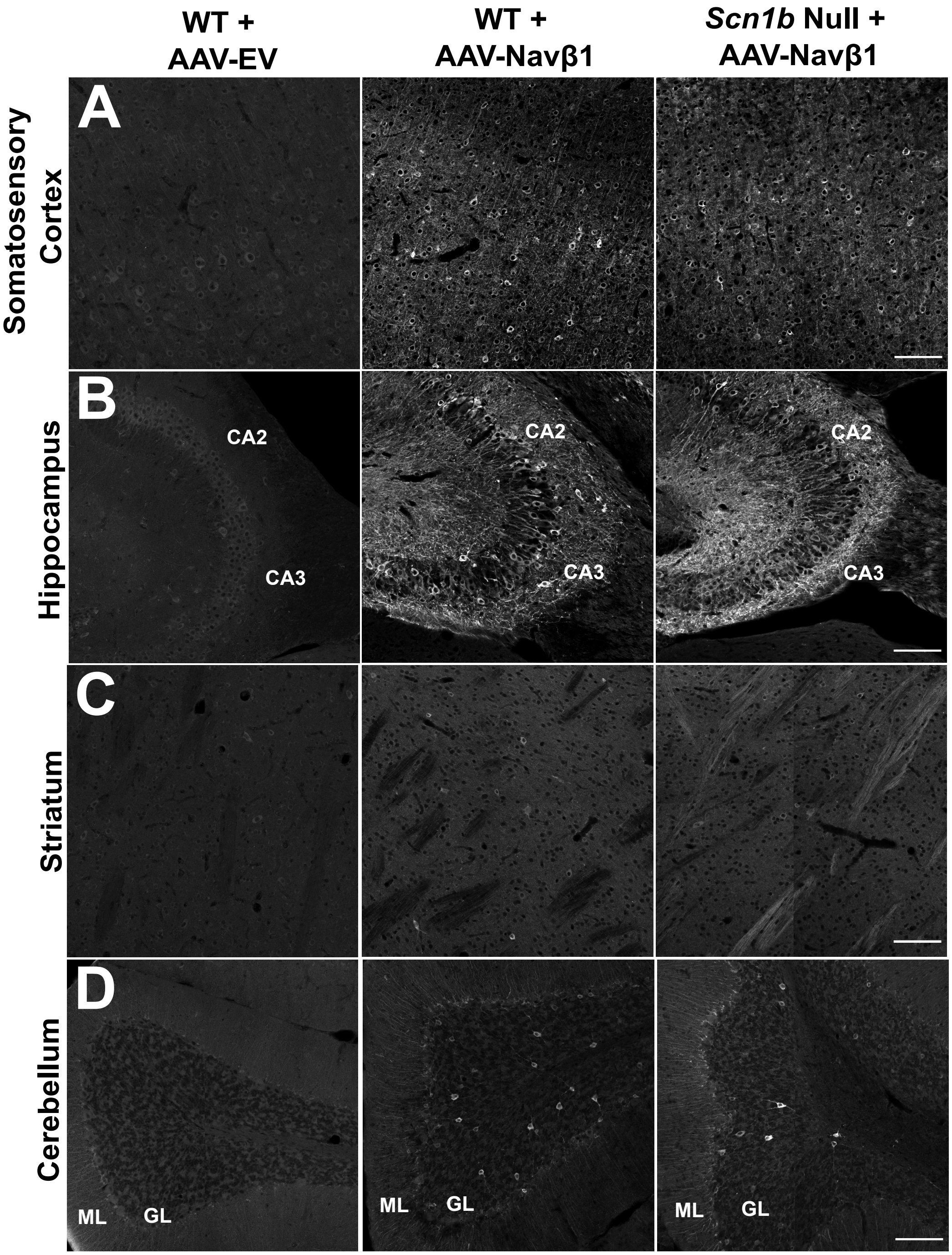

Supplemental Figure 2. Expression of Navβ1-myc in **(A)** the somatosensory cortex, **(B)** the CA2 and CA3 regions of the hippocampus, **(C)** the caudoputamen of the striatum, and **(D)** the cerebellum of P30 WT mice treated with AAV-EV or AAV-Navβ1, and P30 *Scn1b* null mice treated with AAV-Navβ1 at P2. Navβ1-myc expression was detected by immunofluorescence staining with anti-myc antibodySubdivisions of the cerebellar cortex are indicated by ML (molecular layer) and GL (granule cell layer). Scale bars: 100 μm.

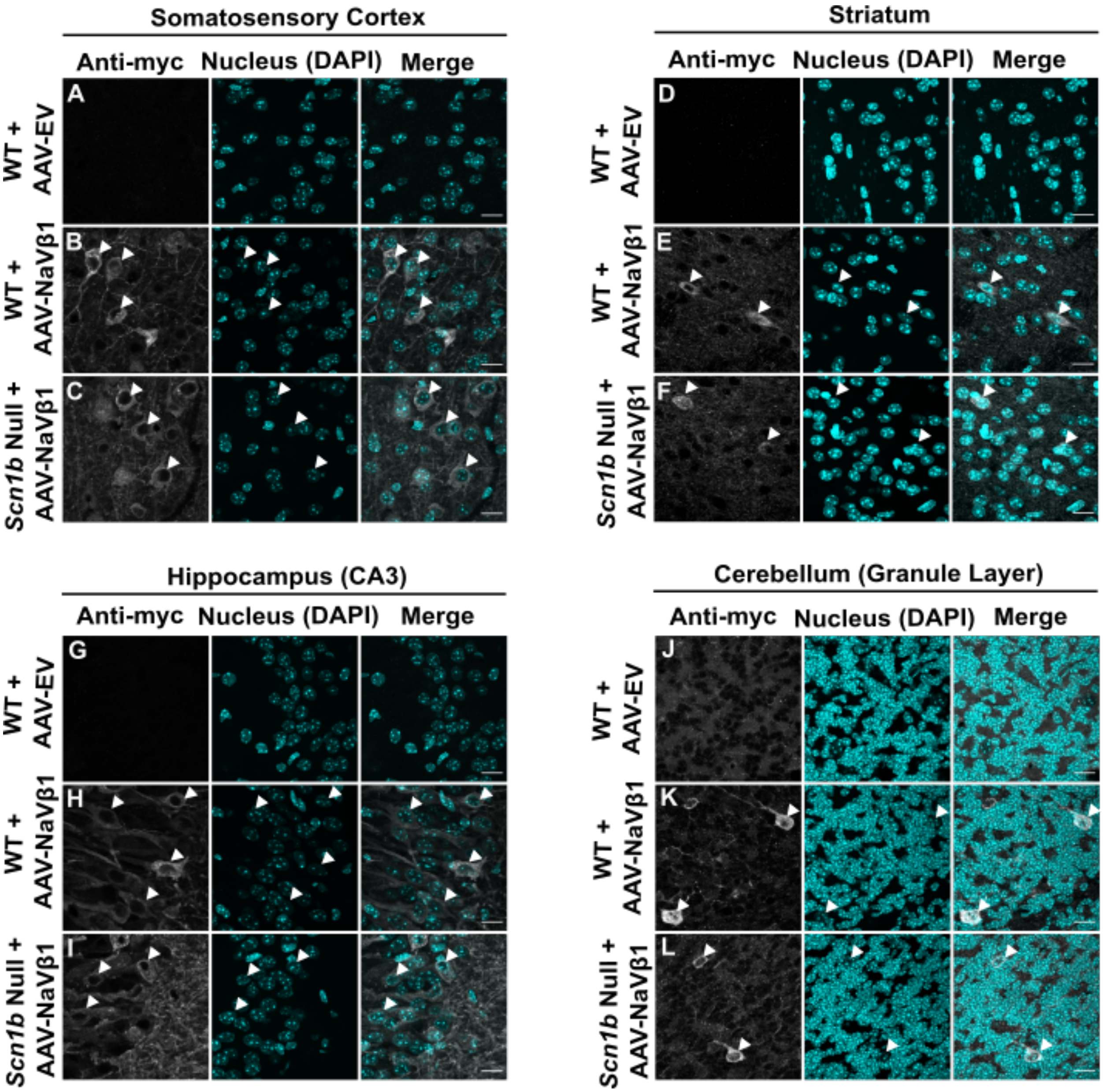

Supplemental Figure 3. High magnification images of Navβ1-myc immunopositive cells (grey) in the somatosensory cortex **(A-C)**, the caudoputamen of the striatum **(D-F)**, the pyramidal cell layer of the hippocampal CA3 region **(G-I)**, and the granule layer of the cerebellum **(J-L)** of P30 WT mice treated with AAV-EV or AAV-β1, and P30 *Scn1b* null mice treated with AAV-Navβ1 at P2. DAPI (cyan) was used as a nuclear marker. Arrows indicate overlap of select Navβ1-myc positive cells with DAPI signal. Scale bars: 15 μm.

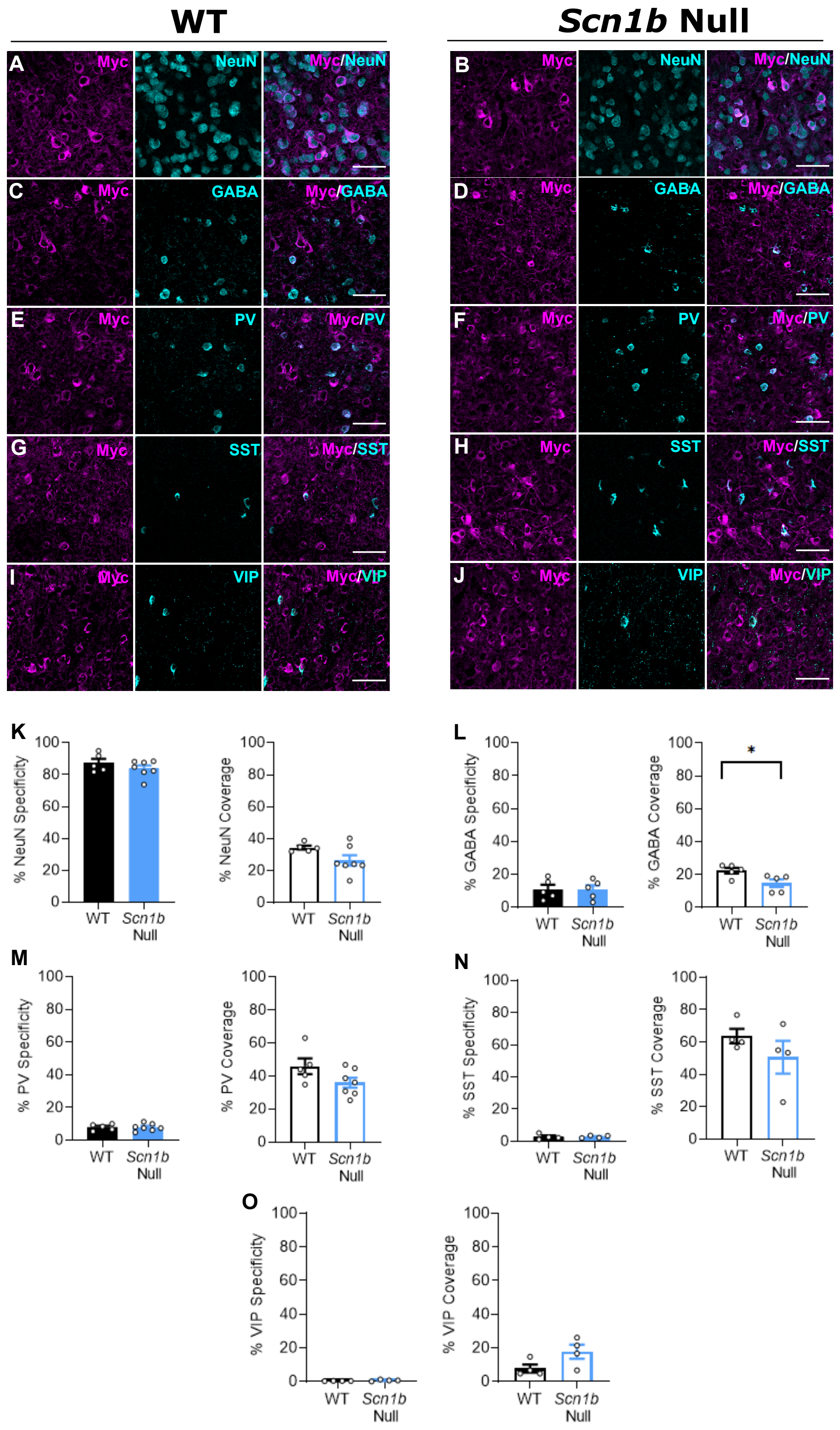

WT
Scn1b Null
A
B
C
D
E
F
G
H
I
J
Myc
NeuN
Myc/NeuN
GABA
Myc/GABA
PV
Myc/PV
SST
Myc/SST
VIP
Myc/VIP
K
L
M
N
O
% NeuN Specificity
% NeuN Coverage
% GABA Specificity
% GABA Coverage
% PV Specificity
% PV Coverage
% SST Specificity
% SST Coverage
% VIP Specificity
% VIP Coverage
*
WT
Scn1b Null

Supplemental Figure 4. Neuronal selectivity of AAV-Navβ1 in somatosensory cortex of WT and *Scn1b* null mice. Expression of Navβ1-myc (magenta) in various cell classes (cyan) in P30 WT **(A, C, E, G, I)** and *Scn1b* null mice **(B, D, F, H, J)**. In panels **K - O**, cell-type specificity was calculated as the number of cells double positive for myc and the cell-type marker divided by the total number of myc positive cells, multiplied by 100%. Cell-type coverage was calculated as the number of cells double positive for myc and the cell-type marker divided by the total number of cell-type marker positive cells, multiplied by 100%. Anti-NeuN was used to label neurons; anti-GABA was used to label GABAergic neurons; anti-PV was used to label fast-spiking PV interneurons; anti-SST was used to label SST expressing interneurons; anti-VIP was used to label VIP expressing interneurons. Scale bars: 50 mm. Quantitative results of NeuN (**K**), GABA (**L**), PV (**M**), SST (**N**) and VIP (**O**) specificity and coverage; N = 4-7 mice in each analytical cohort. * $p < 0.05$, unpaired t-test.

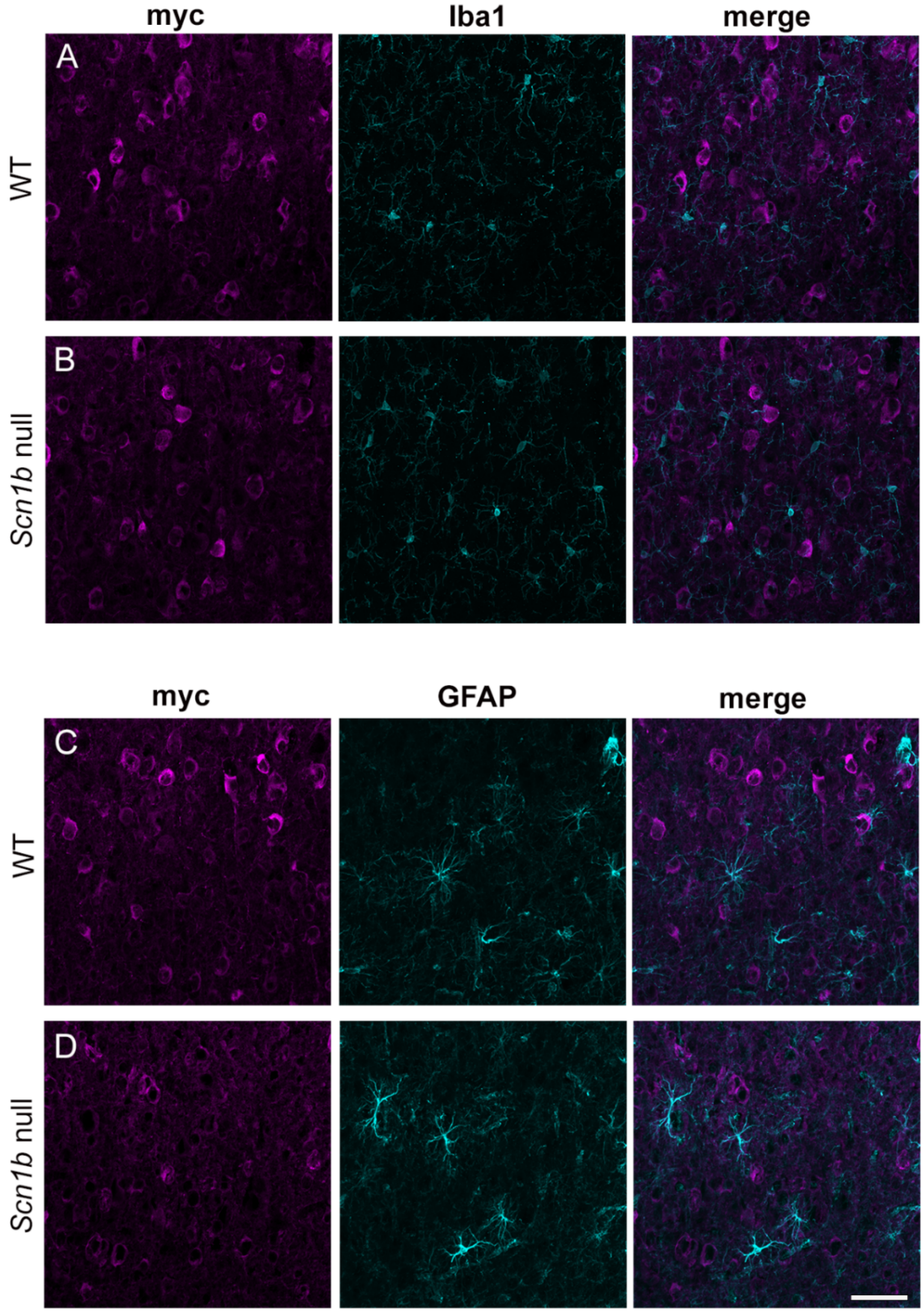

Supplemental Figure 5. Glial expression of Navβ1-myc in somatosensory cortex of P30 WT and *Scn1b* null mice. Navβ1-myc expression is not detected in Iba1+ microglia or GFAP+ astroglia. Navβ1-myc (magenta) expression is not detected in Iba1+ microglia (cyan) in either **(A)** WT or **(B)** *Scn1b* null somatosensory cortex. Navβ1-myc (magenta) expression is not detected in GFAP+ astroglia (cyan) in either **(C)** WT or **(D)** *Scn1b* null somatosensory cortex. Right column: merged images. Scale bar: 50 µm.

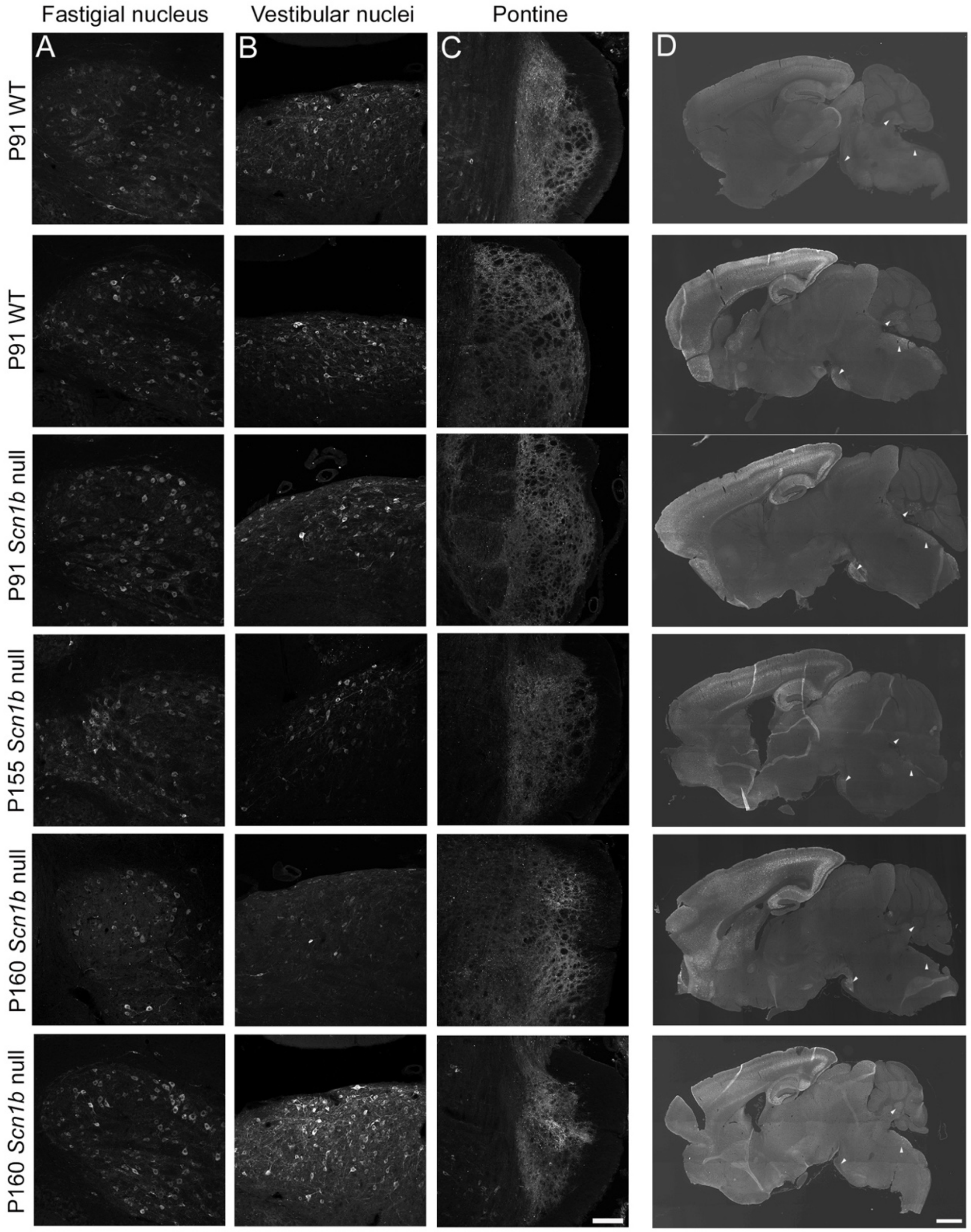

Supplemental Figure 6. Navβ1-myc expression in multiple regions of AAV-Navβ1 injected mice after P90. **(A)** Expression of Navβ1-myc in cerebellar fastigial nucleus. **(B)** Expression of myc-β1 in brainstem vestibular nuclei. **(C)** Expression of Navβ1-myc in brainstem pontine grey. **(D)** Low magnification images showing whole-brain expression of Navβ1-myc, with arrowheads indicating the regions imaged at higher magnification in columns A-C. Scale bar (columns A-C): 100 μm. Scale bar (column D): 1 mm. Genotype and age are indicated on the left side of the figure.

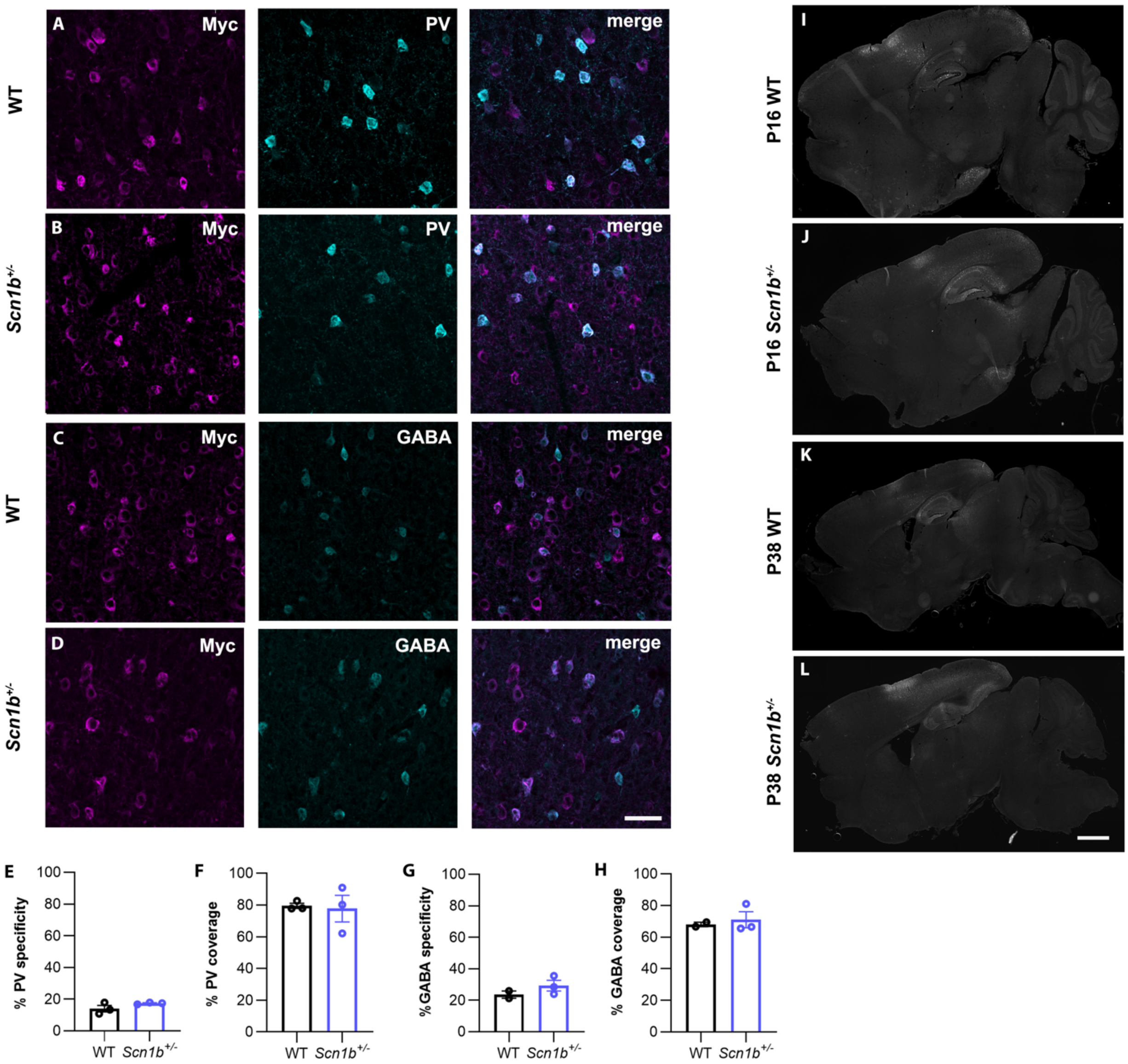

Supplemental Figure 7. **Expression of Navβ1-myc in *Scn1b* mice injected at P10. (A-D)** Neuronal selectivity of AAV-Navβ1 in somatosensory cortex of P38 WT and *Scn1b*$^{+/-}$ mice treated with AAV-Navβ1 at P10. Expression of Navβ1-myc (magenta) in PV+ (**A, B**) or GABA+ (**C, D**) interneurons (cyan) in WT (**A, C**) and *Scn1b*$^{+/-}$ mice (**B, D**). Scale bar: 50 μm. **(E-H)** Quantitative results of PV (**E, F**) and GABA (**G, H**) specificity and coverage expressed as a percentage, where specificity = number of cells double positive for myc and PV or GABA divided by the number of PV+ or GABA+ cells, and coverage = number of double positive cells divided by the number of myc+ cells. N = 3 mice per genotype. **(I-L)** Navβ1-myc expression in whole brain sections. Navβ1-myc expression is primarily restricted to cortex and hippocampus at P16 in WT (**I**) and *Scn1b* null mice (**J**), and at P38 in WT (**K**) and *Scn1b*$^{+/-}$ mice (**L**). Scale bar: 1 mm.

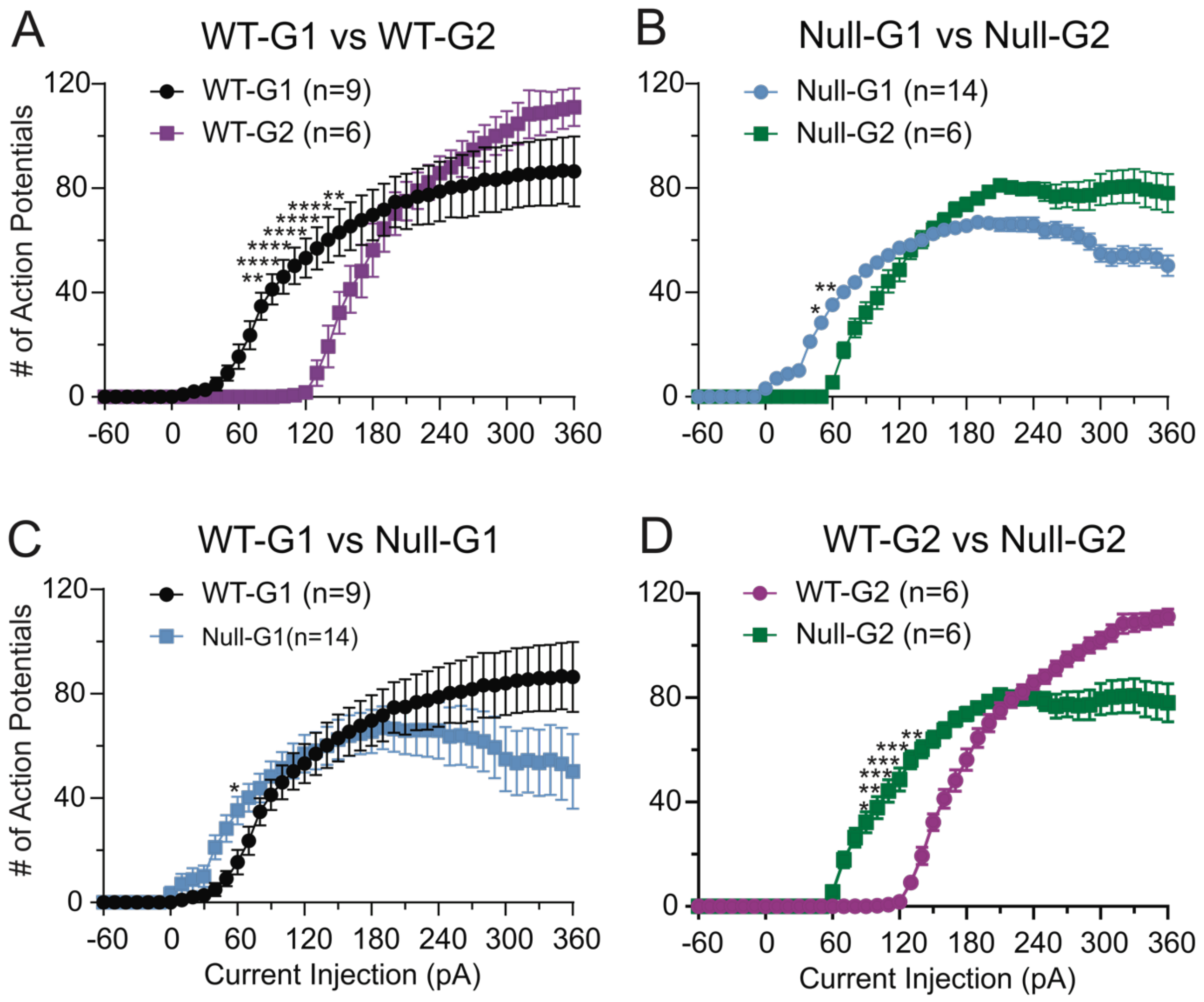

Supplemental Figure 8. **WT and *Scn1b* null mice have two subpopulations of PV+ interneurons in terms of sensitivity to depolarizing current injection**. **(A)** Comparison of I-O curves for AP firing of untreated WT subgroup 1 (black: WT-G1, lower threshold group) and subgroup 2 (purple: WT-G2, higher threshold group). **(B)** Comparison of I-O curves for AP firing of untreated null subgroup 1 (blue: Null-G1, lower threshold group) and subgroup 2 (green: Null-G2, higher threshold group). **(C)** Comparison of I-O curves for AP firing of untreated WT subgroup 1 (black: WT-G1) and null subgroup 1 (blue: Null-G1). **(D)** Comparison of I-O curves for AP firing of untreated WT subgroup 2 (purple: WT-G2) and null subgroup2 (green: Null-G2). Values are mean ± SEM of 6-14 cells (as indicated in the parenthesis) from 7 untreated WT mice or 8 untreated *Scn1b* null mice, respectively.

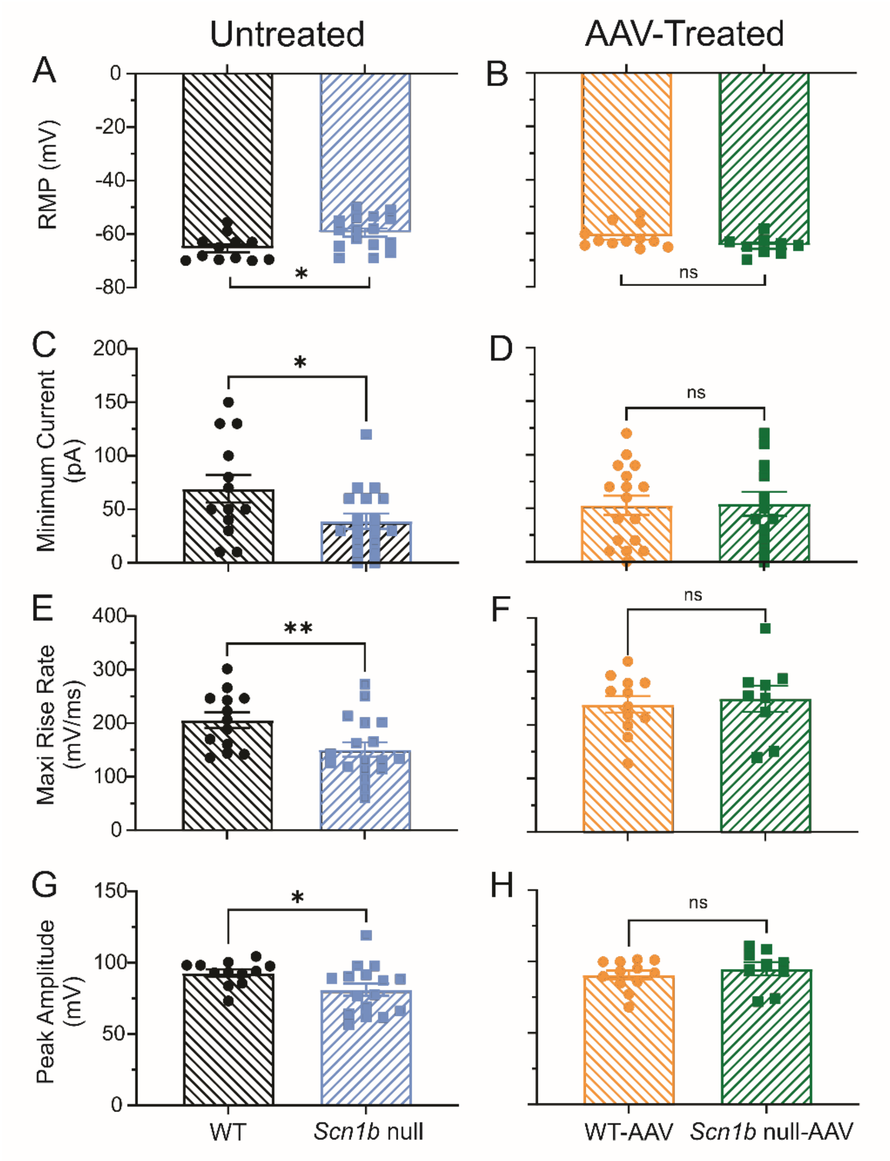

Supplemental Figure 9. AP properties of P16-18 WT vs. *Scn1b* null untreated and AAV-Navβ1-treated PV+ interneurons. (A, B) Comparison of RMP of untreated or AAV-Navβ1-treated WT or *Scn1b* null P16-18 PV+ interneurons, respectively. **(C, D)** Comparison of minimum current required to fire an AP in untreated or AAV-Navβ1-treated WT or *Scn1b* null P16-18 PV+ interneurons, respectively. **(E, F)** Comparison of maximum rise rates of APs of untreated or AAV-Navβ1-treated WT or *Scn1b* null P16-18 PV+ interneurons, respectively. **(G, H)** Comparison of peak AP amplitude of untreated or AAV-Navβ1-treated WT or *Scn1b* null P16-18 PV+ interneurons, respectively. Values are mean ± SEM of 12-13 cells from 6-7 untreated WT mice, 12-14 cells from 4-6 AAV-treated WT mice, 16-18 cells from 7 untreated *Scn1b* null mice, or 9-12 cells from 4-6 AAV-treated *Scn1b* null mice. Untreated WT: black; untreated *Scn1b* null: blue; AAV-Navβ1-treated WT: orange; AAV-Navβ1-treated *Scn1b* null: green. * $p<0.05$, ** $p<0.01$. Results are tabulated in Supplemental Table 1.

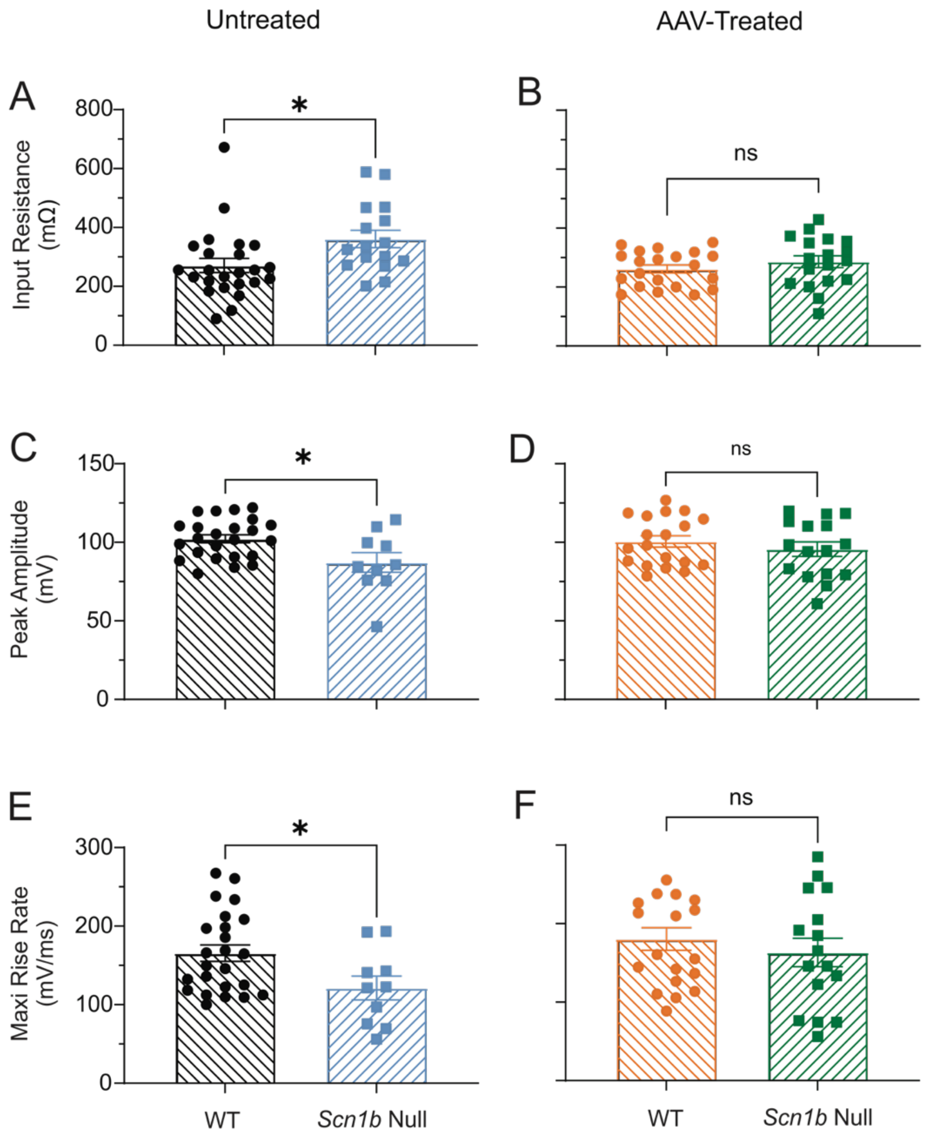

Supplemental Figure 10. AP properties of P16-18 WT vs. ***Scn1b*** **null untreated and AAV-Navβ1-treated cortical layer 2-6 pyramidal neurons. (A, B)** Comparison of input resistance of untreated or AAV-Navβ1-treated WT or *Scn1b* null P16-18 pyramidal neurons, respectively. **(C, D)** Comparison of peak AP amplitude in untreated or AAV-Navβ1-treated WT or *Scn1b* null P16-18 pyramidal neurons, respectively. **(E, F)** Comparison of maximum rise rates of APs of untreated or AAV-Navβ1-treated WT or *Scn1b* null P16-18 pyramidal neurons, respectively. Values are mean ± SEM of 22-24 cells from 6-7 untreated WT mice, 12-21 cells from 4-6 AAV-treated WT mice, 9-12 cells from 7 untreated *Scn1b* null mice, or 14-18 cells from 4-6 AAV-treated *Scn1b* null mice. Untreated WT: black; untreated *Scn1b* null: blue; AAV-Navβ1-treated WT: orange; AAV-Navβ1-treated *Scn1b* null: green. * $p<0.05$, ** $p<0.01$. Results are tabulated in Supplemental Table 2.

Supplemental Tables

Parameters	WT<br>untreated	*Scn1b* null<br>untreated	WT-AAV	*Scn1b* null-AAV
Resting Membrane Potential<br>mV	-78.38 ± 1.38<br>(12)	**-72.44 ± 1.56***<br>(25)	-73.73 ± 1.12<br>(17)	-77.36 ± 0.93<br>(11)
Capacitance<br>pF	16.66 ± 1.37<br>(14)	15.61 ± 2.36<br>(15)	13.64 ± 1.17<br>(12)	13.56 ± 1.23<br>(9)
Input Resistance<br>mΩ	194.32 ± 11.35<br>(14)	**241.44 ± 17.04***<br>(16)	265.97 ± 26.43<br>(13)	243.19 ± 20.46<br>(12)
Threshold Potential<br>mV	-55.84 ± 2.67<br>(11)	-49.08 ± 3.09<br>(15)	-50.73 ± 2.64<br>(12)	-58.16 ± 3.02<br>(9)
Peak AP Amplitude<br>mV	92.8 ± 2.45<br>(12)	**80.51 ± 4.53***<br>(15)	90.68 ± 2.98<br>(12)	94.97 ± 4.61<br>(9)
Half-Width<br>ms	1.39 ± 0.17<br>(12)	1.44 ± 0.15<br>(15)	0.93 ± 0.05<br>(12)	1.00 ± 0.07<br>(9)
Maximum Rise Rate<br>mV/ms	210.83 ± 15.10<br>(12)	**157.47 ± 15.38****<br>(15)	238.65 ± 15.63<br>(12)	249.02 ± 24.38<br>(9)
Maximum Decay Rate<br>mV/ms	-75.94 ± 6.59<br>(12)	-67.50 ± 5.43<br>(15)	-113.98 ± 6.64<br>(12)	-107.08 ± 10.85<br>(9)

Supplemental Table 1. Comparisons of passive and active membrane electrical properties of cortical layer 2-6 WT and *Scn1b* null PV+ interneurons with or without AAV-Navβ1 treatment. All values are expressed as mean ± SEM (n). * $p<0.05$; ** $p<0.01$, compared with untreated WT, Student's unpaired *t* test or Mann-Whitney test.

**Parameters**	**WT untreated**	***Scn1b* null untreated**	**WT-AAV**	***Scn1b* null-AAV**
Resting Membrane Potential mV	-75.98 ± 1.26 (24)	-75.35 ± 2.12 (10)	-73.43 ± 1.72 (18)	-76.00 ± 1.57 (16)
Capacitance (pF)	15.00 ± 1.59 (23)	12.92 ± 1.75 (12)	14.91 ± 1.88 (12)	12.45 ± 1.41 (16)
Input Resistance (mΩ)	254.43 ± 18.12 (22)	**341.07 ± 28.61*** (14)	261.58 ± 13.09 (21)	286.31 ± 20.21 (18)
Threshold Potential (mV)	-44.26 ± 1.12 (24)	-44.17 ± 1.56 (9)	-46.42 ± 1.84 (19)	-44.24 ± 1.53 (16)
Peak AP Amplitude (mV)	102.8 ± 2.56 (24)	**87.21 ± 6.23*** (10)	100.59 ± 3.60 (19)	95.69 ± 4.61 (16)
Half-Width (ms)	3.54 ± 0.27 (24)	3.73 ± 0.42 (10)	2.87 ± 0.23 (18)	2.90 ± 0.07 (14)
Maximum Rise Rate (mV/ms)	165.66 ± 10.46 (24)	**121.22 ± 15.16*** (10)	180.47 ± 14.39 (19)	163.35 ± 18.13 (16)
Maximum Decay Rate (mV/ms)	-33.52 ± 2.31 (24)	-29.91 ± 2.67 (10)	-39.48 ± 3.84 (19)	-38.95 ± 5.19 (14)

Supplemental Table 2: Comparisons of passive and active membrane electrical properties of cortical layer 2-6 WT and *Scn1b* null pyramidal cells with or without AAV-Navβ1 treatment. All values are expressed as mean ± SEM (n). * $p<0.05$; ** $p<0.01$, compared with untreated WT*,* Student's unpaired *t* test or Mann-Whitney test.

Supplemental Movie

Supplemental Movie 5: This movie consists of 2 video clips. The first video clip shows 3 P100 AAV-Navβ1-treated *Scn1b* null mice. The second video clip shows 2 P101 AAV-Navβ1-treated *Scn1b* null mice plus 1 AAV-Navβ1-treated P101 WT mouse from the same litter for size comparison.
