# Supplementary figures and images for: Neonatal but not juvenile gene therapy reduces seizures and prolongs lifespan in SCN1B–Dravet syndrome mice
Source: J Clin Invest. 2025 Jan 23;135(5):e182584. doi: 10.1172/JCI182584 (PMC11870736; doi:10.1172/JCI182584)

Original, uncropped blots shown in Figure 1

Figure 1, panel J

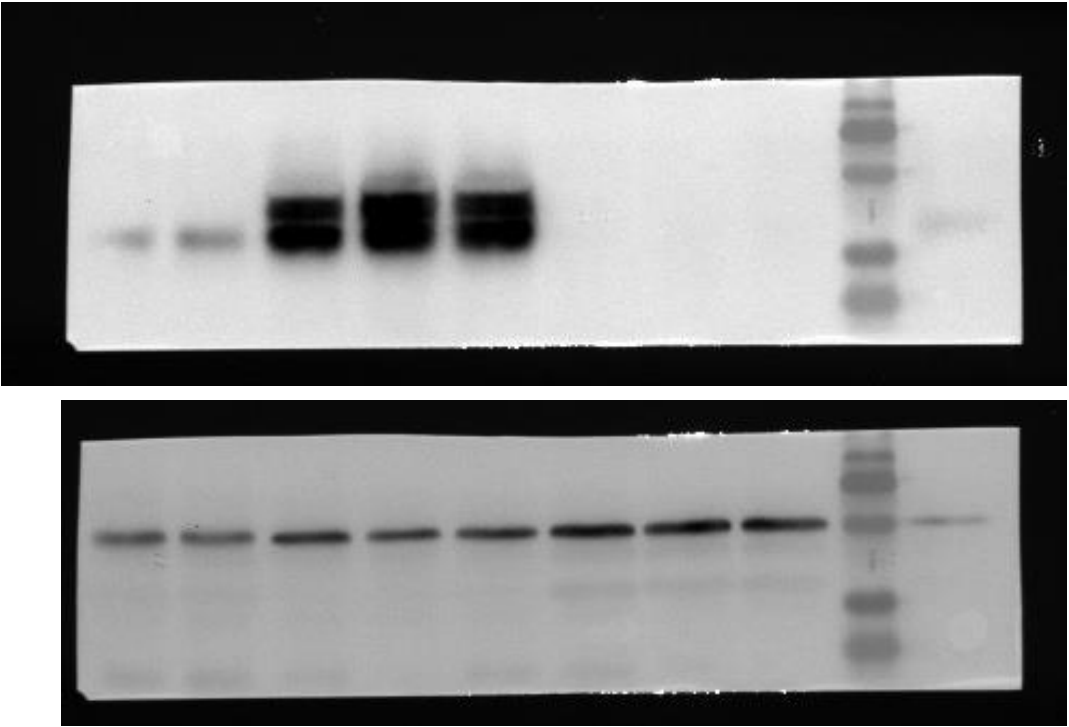

Figure 1, panel K

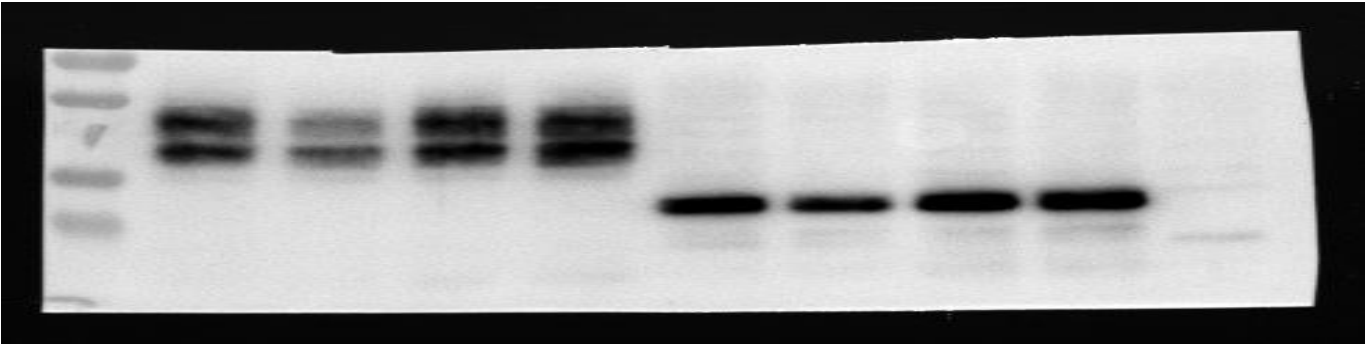

Supplement: Unedited blot and gel images [file jci-135-182584-s232.pdf]
